# Genetic and Clinical Analyses of the *KIZ*-c.226C>T Variant Resulting in a Dual Mutational Mechanism

**DOI:** 10.3390/genes15060804

**Published:** 2024-06-18

**Authors:** Yogapriya Sundaresan, Antonio Rivera, Alexey Obolensky, Prakadeeswari Gopalakrishnan, Hanit Ohayon Hadad, Aya Shemesh, Samer Khateb, Maya Ross, Ron Ofri, Sharon Durst, Hadas Newman, Rina Leibu, Shiri Soudry, Dinah Zur, Tamar Ben-Yosef, Eyal Banin, Dror Sharon

**Affiliations:** 1 Department of Ophthalmology, Hadassah Medical Center, Faculty of Medicine, Hebrew University of Jerusalem, Jerusalem 91120, Israel; yogapriya.sundar@mail.huji.ac.il (Y.S.); antonioriveravasq@gmail.com (A.R.); prakadee.gopalakrish@mail.huji.ac.il (P.G.); banine@mail.huji.ac.il (E.B.); 2 Koret School of Veterinary Medicine, The Hebrew University of Jerusalem, Rehovot 76100, Israel; 3 Ophthalmology Division, Tel Aviv Sourasky Medical Center, Affiliated to Faculty of Medical & Health Sciences, Tel Aviv University, Tel Aviv 69978, Israel; 4 Department of Ophthalmology, Rambam Health Care Center, Haifa 31096, Israel; 5 Department of Ophthalmology, Rabin Medical Center, Petah Tikva 49100, Israel; 6 The Ruth & Bruce Rappaport Faculty of Medicine, Technion-Israel Institute of Technology, Haifa 31096, Israel

**Keywords:** clinical manifestation, exonic sequence enhancer, gene expression, retinal disease

## Abstract

Retinitis pigmentosa (RP) is a heterogeneous inherited retinal disorder. Mutations in *KIZ* cause autosomal recessive (AR) RP. We aimed to characterize the genotype, expression pattern, and phenotype in a large cohort of *KIZ* cases. Sanger and whole exome sequencing were used to identify the *KIZ* variants. Medical records were reviewed and analyzed. Thirty-one patients with biallelic *KIZ* mutations were identified: 28 homozygous for c.226C>T (p.R76*), 2 compound heterozygous for p.R76* and c.3G>A (p.M1?), and one homozygous for c.247C>T (p.R83*). c.226C>T is a founder mutation among patients of Jewish descent. The clinical parameters were less severe in *KIZ* compared to *DHDDS* and *FAM161A* cases. RT-PCR analysis in fibroblast cells revealed the presence of four different transcripts in both WT and mutant samples with a lower percentage of the WT transcript in patients. Sequence analysis identified an exonic sequence enhancer (ESE) that includes the c.226 position which is affected by the mutation. *KIZ* mutations are an uncommon cause of IRD worldwide but are not rare among Ashkenazi Jews. Our data indicate that p.R76* affect an ESE which in turn results in the pronounced skipping of exon 3. Therefore, RNA-based therapies might show low efficacy since the mutant transcripts are spliced.

## 1. Introduction

Inherited retinal diseases (IRDs) are a heterogenous group of ocular disorders characterized by the degeneration and dysfunction of photoreceptor cells leading to vision impairment. There are more than 50 major types of IRDs, among which retinitis pigmentosa (RP, OMIM 268000) is considered one of the most genetically and clinically heterogeneous diseases in humans [1,2,3,4]. Population-based analysis indicated that 1 in 4000 individuals are affected by RP worldwide [5]. RP is characterized by significant variability among patients [6,7,8], with many exhibiting the classic symptoms such as impaired dark adaptation and night blindness during adolescence and loss of mid-peripheral visual field during young adulthood, both attributed to rod dysfunction [9]. As the disease progresses, patients suffer the loss of far peripheral vision, eventually developing tunnel vision, and ultimately experiencing the compromise of the central vision due to cone impairment in the later stages of life [5]. 

The ocular phenotype of RP follows a Mendelian pattern of inheritance where 15–25% are autosomal dominant, 35–50% are autosomal recessive (AR), and 7–15% are X-linked [10]. Pathogenic variants in over 60 genes are responsible for RP [1]. A unique group of genes that are associated with IRDs are those encoding proteins that are involved in the morphogenesis and function of the photoreceptor sensory cilia, a microtubule-based organelle coordinating various cellular processes [11,12,13]. Pathogenic variants in cilia-related genes result in retinal ciliopathies including Leber congenital amaurosis (LCA), RP, macular degeneration, cone-dystrophy, and cone-rod dystrophy [14]. Among these genes, *KIZ* was reported in 2014 to cause ARRP due to biallelic pathogenic variants [15]. To date, only seven additional publications reported biallelic *KIZ* mutations in IRD patients (Table S1) [16,17,18,19,20,21,22,23,24]. The most commonly-reported *KIZ* pathogenic variant is c.226C>T (p.R76*), a nonsense mutation leading to a premature termination codon (PTC) in exon 3. This variant has been reported in 18 patients with biallelic mutations, among whom seven subjects were previously included in a comprehensive analysis of IRD cases reported by us [20]. The current study will focus on further investigating this particular variant. For eight additional patients, clinical analysis was reported [15,16,22], and is largely in-line with the phenotypic spectrum of ARRP. The *KIZ* gene encodes the centrosomal protein kizuna that is expressed in the primary cilia and functions by stabilizing as well as strengthening the pericentriolar region prior to spindle formation, thus playing a vital role in cell cycle progression [25]. The human *KIZ* gene shows high expression levels in the retina, while lower levels were detected in the retinal pigment epithelium (RPE), fibroblast cell-lines, and white blood cells [15]. In addition, transcriptomic analysis revealed the presence of the mouse ortholog (Plk1s1) in rod photoreceptors and reduced Plkls1 expression was evident in a rd1 mouse model exhibiting photoreceptor degeneration [15,17]. 

The current study includes clinical information on 31 RP patients harbouring biallelic *KIZ* mutations, the most common of which is c.226C>T (p.R76*) identified in 58 of the 62 (93.5%) mutated alleles. In addition, through gene expression analysis conducted on skin fibroblasts of WT individuals and patients carrying this mutation (c.226C>T), we provide evidence suggesting a dual mutational mechanism that potentially impacts the efficiency of RNA-based treatment modalities.

## 2. Materials and Methods

Please see Supplementary Materials for a full description of the methods used in this study.

### 2.1. Recruitment and Clinical Analyses

Participants were recruited from Hadassah Medical Center, Tel Aviv Medical Center, and Rambam Medical Center, Israel. Ethical approval was obtained at individual Institutional Review Boards. The tenets of the Declaration of Helsinki were followed. Participants provided written informed consent after receiving an explanation about the study and its possible consequences before donating the blood sample. The ophthalmic evaluation included a full ophthalmological examination, Goldmann perimetry (using Humphrey or Octopus systems), full field electroretinography (FFERG) according to the ISCEV standard [26] (using LKC or Diagnosys systems), colour vision testing using the Ishihara 38-panel and Farnsworth–Munsell D-15 tests, and colour, autofluorescence and OCT imaging using Optos Silverstone, Optos California, TOPCON, Eidon and Heidelberg Spectralis systems. The clinical assessment of the *KIZ* patients encompassed visual acuity (VA) testing (25 cases), refraction (12), visual fields (10), funduscopy (15), optical coherence tomography (OCT-12), electroretinography (ERG-14), and electro-oculography (EOG-6). 

### 2.2. Statistical Analysis

A principal component analysis (PCA) of the clinical parameters was performed using RStudio. PCA is a dimensionality reduction method that can translate n variables into n orthogonal linear combinations of those variables. Those linear combinations preserve the total variance. The first linear combination, PC1, is the linear combination with the greatest sample variance among all n possible linear combinations. The second principal component (PC2) is defined as the linear combination that accounts for a maximal proportion of the remaining variance subject to being uncorrelated with the first principal component and so on. In the current study, we analysed the following seven variables by PCA: disease onset age, age of presentation, rod response, cone flicker amplitude, a-wave cone-rod response, b-wave cone-rod response, and slope of best-corrected visual acuity (BCVA) with age. The Tukey multiple comparisons of means analysis was used to examine the difference between each group to all other groups in a pair-wise comparison.

### 2.3. Genetic Analyses

DNA was extracted by the Maxwell DNA purification kit (Promega, Madison, WI, USA). Whole exome sequencing (WES) was performed on genomic DNA samples at Pronto Diagnostics Inc. (Tel Aviv, Israel) using Agilent SureSelect Human All Exon V4 kit, Variantyx Ltd. (Framingham, MA, USA) using Agilent SureSelect v6 on NovaSeq Illumina, and 3 billion using IDT xGen Exome Research Panel V2. The alignment of the NGS fastq files was performed using the Galaxy platform (https://galaxyproject.org, accessed on 18 June 2024). The reads were initially aligned to hg19 using HISAT2 aiming to identify the transcripts present in the analysis, followed by Bowtie2 alignment to a custom-made fasta reference file containing the different transcripts. BAM files were viewed by IGV and the number of reads representing each transcript was tabulated. Variant files were annotated using ANNOVAR according to the dbSNP database as well as using the Genoox platform (https://franklin.genoox.com/, accessed on 18 June 2024) with the following filtering steps: (1) Variants in known IRD genes that are located within homozygous regions were analysed prior to any other analysis; (2) All variants in known IRD genes (based on RetNet https://sph.uth.edu/retnet/, accessed on 18 June 2024) were analysed; (3) Variant type: Missense, nonsense, splice-site, stop-loss, insertions, and deletions in the coding region were included; (4) Variants with minor allele frequency (MAF) greater than 1.0% in the gnomAD database (https://gnomad.broadinstitute.org, accessed on 18 June 2024) were excluded; (5) The prediction of the possible effect of each variant was analysed by 3 prediction online programs SIFT (http://sift.jcvi.org/, accessed on 18 June 2024), MutationTaster (https://www.mutationtaster.org/, accessed on 18 June 2024), and PolyPhen2 (http://genetics.bwh.harvard.edu/pph2/, accessed on 18 June 2024). All the suspected pathogenic variants were validated by Sanger sequencing of PCR products. The cDNA was synthesized using the qPCRBIO cDNA Synthesis Kit (PCRBIOSYSTEMS). The RT-PCR products were treated by ExoSAP and underwent library preparation for NGS based on commercial recommendations (Illumina, San Diego, CA, USA).

### 2.4. Extraction of RNA and cDNA Synthesis

The total RNA was isolated from the confluent fibroblast cells using the RNeasy mini kit (Qiagen, Hilden, Germany). Following RNA isolation, cDNA was synthesized using the qScript™cDNA synthesis kit (Quantabio, Beverly, MA, USA). The total RNA was also isolated from the WT mouse retina (C57B6/J, 1–2 months old) and WT sheep retina (6 months old) that were placed in RNAlater until processing. The RNA was isolated using a Trizol (TRI-reagent)-based RNA extraction method immediately followed by DNase treatment (Thermofisher Scientific, Waltham, MA, USA) and the cDNA was synthesized using the QuantaBio cDNA synthesis kit.

### 2.5. RT-PCR and Next Generation Sequencing (NGS)

The following primers were designed using Primer3 (https://bioinfo.ut.ee/primer3-0.4.0/, accessed on 18 June 2024) to amplify the human *KIZ* fragment including exons 2 to 5 by RT-PCT: Forward primer-5′-TCGTCGGCAGCGTCAGATGTGTATAAGAGACAG aaagaagagattggacctgg-3′, Reverse primer-3′-GTCTCGTGGGCTCGGAGATGTGTATAAGAGACAG ggctatcttttgagcatagc-5′. The uppercase letters represent NGS adapters, and the lowercase letters represent the *KIZ* specific sequence. The designed primer sequences were linked to a universal adapter sequence to facilitate the indexing of each sample before NGS. RT-PCR was carried out using 2X PCRBIO Taq mix red (PCR Biosystems, London, UK). The products were loaded on an agarose gel to visualise the expression pattern of *KIZ*. In addition, gel densitometry analysis was carried out using ImageJ software (Version number 1.53k). Further, the RT-PCR products were treated by ExoSAP followed by either Sanger sequencing or library preparation for NGS that was based on commercial recommendations (Illumina, San Diego, CA, USA). The single-reads (reads length of 150 bp) were sequenced using Illumina NextSeq500 with an average of >100,000 reads per sample. Following sequencing, the NGS fastq files were aligned using Usegalaxy (https://galaxyproject.org, accessed on 18 June 2024). The files were uploaded to the program and initially groomed using Fastq Groomer followed by Bowtie2 alignment to a custom-made fasta reference file containing the different transcripts. The generated BAM files were viewed by IGV and the number of reads representing each transcript was tabulated. Further, the percentage of reads corresponding to each transcript was calculated. The following primer pair was used to amplify both the mouse and sheep *KIZ* fragment (453 bp including the NGS adapters) by RT-PCR: *KIZ* F-5′-AAAAGAAGAGATTGGACCTG-3′ and *KIZ* R-5′-CAACAATCTTTATGGGCCGC-3′. The *PGK* (NM_008828.3) expression was used as an endogenous control with the following primers: *Pgk1*-E3F-5′-GATCAAGGCTGCTGTTCCA and Pgk1-E5R-5′-GCAGTCCCAAAAGCATCATT.

### 2.6. Exonic Splicing Enhancer Sequence (ESE) Analysis 

Putative ESE sequences in exon 3 of the control sequence were identified using the online program ESEfinder 3.0 (http://krainer01.cshl.edu/cgi-bin/tools/ESE3/esefinder.cgi, accessed on 18 June 2024) [27]. Each predicted ESE sequence was recognized by a Serine/Arginine-rich splicing factor (SRSF) and the tool provides a score for each suspected ESE. Based on the inbuilt algorithm, the thresholds obtained for SRSF1, SRSF2, SRSF5, and SRSF6 are 1.956, 2.38, 2.67, and 2.676 respectively. Predicted sequences holding a threshold higher than the above-mentioned values are suggested to be a potential ESE sequence. The present study has included only the highest scored ESE sequence including the c.226 position. In addition, the mutant sequence was also fed into the program to determine if the mutation had an effect on the potential ESE by projecting a score below the threshold.

## 3. Results

### 3.1. Clinical Evaluation of KIZ Patient 

Our cohort of IRD cases within the Israeli and Palestinian populations comprises 839 families affected by RP (with 1163 recruited patients). Using various genetic analysis tools (such as Sanger sequencing of founder mutations, whole exome sequencing-WES, and mutation panels), we identified 30 families with RP (Table 1 and Figure S1), including 31 RP patients harbouring biallelic pathogenic variants in *KIZ*. In addition, we have identified three other siblings affected by RP who have not yet been recruited to this study. The most common mutation was c.226C>T (p.R76*) that we identified homozygously in 27 families (28 patients) and heterozygously in two families (2 patients) in trans with c.3G>A (p.M1?). Both mutations have been previously reported (Table S1). In addition, we identified one index case who was homozygous for a novel variant, c.247C>T (p.R83*). 

With an objective to study the retinal clinical features associated with *KIZ*-related RP, we collected clinical data from 25 patients (Table S2). No consistent involvement of organs other than the eye was identified in this cohort, suggesting that *KIZ* is associated predominantly with nonsyndromic RP. 

**Figure 1:**
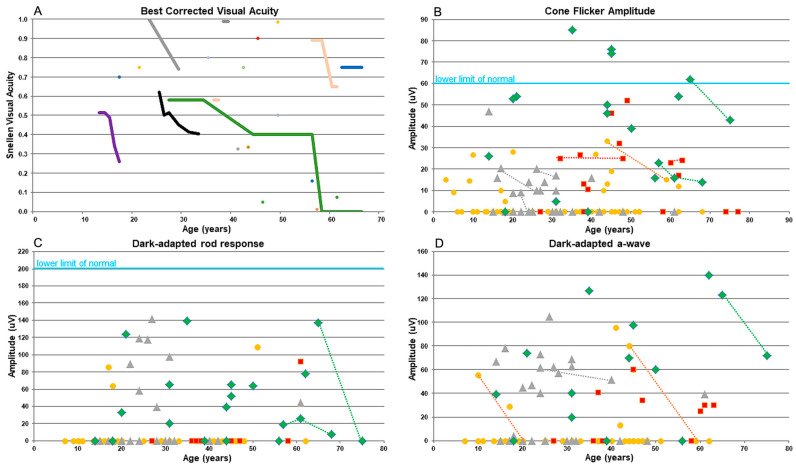
Graphs showing the decline of visual acuity (**A**) and ERG responses (**B**–**D**) versus age (years) in *KIZ* patients. (**A**) Snellen visual acuity. The colours represent data collected from different patients. For some patients, only a single measurement of VA was available and is represented by a single value, while for other patients, multiple measurements were available and were connected by lines. (**B**) Cone photoreceptor function as measured in response to a white 30-Hz flicker stimulus versus age (lower limit of normal cone 30-Hz flicker ERG amplitudes, 60 uV). (**C**) Dark-adapted rod response. (**D**) Dark-adapted a-wave. Each point represents the average of both eyes versus age. Data from cases with multiple data points are connected with a line. Data were collected from patients with biallelic mutations in *KIZ* (green), *MAK* (red), *DHDDS* (grey), and *FAM161A* (yellow).

**Figure 2:**
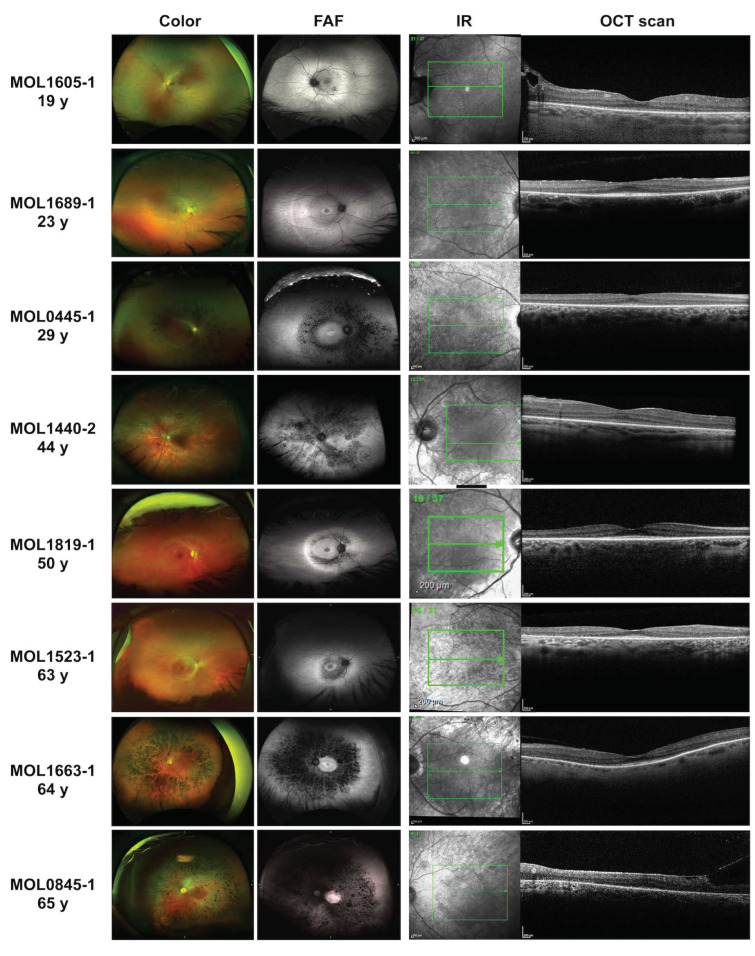
Ultra-wide field pseudocolour, fundus autofluorescence (FAF), infra-red (IR), and optical coherent tomography (OCT) imaging in patients with retinitis pigmentosa (RP) resulting from *KIZ* mutations at different ages. See detailed description in the Supplementary Materials. The age is represented in years (y).

The VA metrics collected in 26 *KIZ* subjects showed that most patients maintain relatively good vision through the 6th decade of life. Up to the age of 50 years, all patients but one had VA > 0.3 and two patients above the age of 60 years had VA > 0.6 (Table S2 and Figure 1A). All patients exhibited unremarkable anterior segments and any cataractous changes were mild: notably, the youngest patient with a cataract was 29 years old. In our cohort, only two patients underwent cataract surgery, at the ages of 44 and 56 years. Data on refractive error was available for 12 patients with variable values ranging from myopic to hyperopic correction along with variable astigmatism (Table S2). Among our cohort, the visual fields (n = 10) were affected relatively early in the course of the disease, and visual field testing showed the progression from multiple scotomas to the severely restricted, tunnel vision typical for advanced RP. Fundus findings ranged from very mild to a typical RP-like appearance (Figure 2). Seven patients had minimal funduscopic changes which included subtle attenuation of the retinal vessels and fine mottling of the RPE. On fundus autofluorescence (FAF) of these eyes, hypoautofluorescent spots and patches surrounding the arcades along with a hyperautofluorescent ring in the macular area were noted. One of the patients (MOL1605-1 in Figure 2) had a pigmented lesion, which appeared to be not related to the rest of the findings, and the position close to the optic nerve seemed to not affect the VA as well as the retinal architecture. Eight other patients exhibited retinal findings consistent with RP, including bone spicule-like pigmentation, vessels attenuation, and a waxy pallor of the optic disc. These changes were more easily discernible on FAF imaging accompanied by a hyperautofluorescent ring in the macular region. OCT was performed on 12 patients demonstrating a reduction in the outer nuclear layer (ONL) thickness and a small island of ellipsoid zone (EZ) preservation in the fovea (Figure 2). Six patients presented with an epiretinal membrane (ERM) which did not appear to affect their vision. Two patients had cystoid macular oedema (CME) with a good response to carbonic anhydrase inhibitors.

Full-field ERG (ffERG) was performed in 14 patients (Figure 1B–D), revealing a pattern consistent with rod-cone dystrophy. Initially, at the first examination, most patients exhibited measurable amplitudes which remained recordable even at the age of 75 years. Despite the persistence of measurable amplitudes, there were observable alterations in the ERG responses. Specifically, the cone flicker responses exhibited relatively preserved amplitudes but delayed implicit times (Figure 1B), while the rod responses were either reduced or completely extinguished (Figure 1C,D), consistent with the diagnosis of rod-cone dystrophy in these patients. 

**Figure 3:**
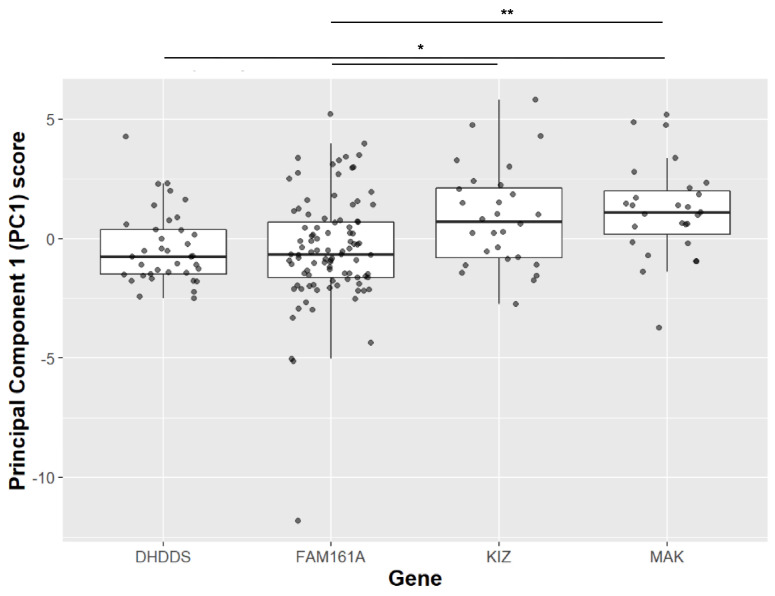
PCA1 analysis of clinical parameters collected from patients harbouring mutations in four IRD-causing genes. Since in all PC1 variables low levels reflect a more severe disease, and since their coefficient are positive scalars (see column 1 in Table S4), one can refer to high patient scores (*Y*-axis) as indicating a more severe disease. Post hoc analysis comparing each pair of genes, showing a significant difference between *MAK* and *DHDDS* (*p*-value = 0.017), *KIZ* and *FAM161A* (*p*-value = 0.018), *MAK* and *FAM161A* (*p*-value = 0.002). * represents *p*-values between 0.01 and 0.05; ** represents a *p*-value < 0.01.

To derive insight on the similarity between *KIZ*-related RP and other prevalent variants, we performed a comparative analysis. Pairwise *t*-test analysis comparing VA and ERG responses of patients with *KIZ* mutations to previously described cases harbouring mutations in *DHDDS*, *FAM161A*, or *MAK* [28,29], revealed that *KIZ* patients have on average higher cone and mixed ERG responses, while the *MAK* group showed higher age of onset and better VA compared to the other three groups (Figure S2). We subsequently performed a principal component analysis (PCA) on the following clinical variables: disease onset age, age of presentation, rod response, cone flicker amplitude, a-wave and b-wave cone-rod responses, and the slope of best-corrected VA (BCVA) with age. The sample variances and the variable coefficient of PC1–7 are shown in Tables S3 and S4. The analysis revealed that PC1 accounts for 67.3% of the variability whereas PC2 explains only 12.9% of the remaining variability. The subsequent principal components (PC3–PC7) accounted for a variability ranging between 0.3% to 8.9%. Therefore, we used PC1 values as a measure of disease severity to compare between four gene-based groups (Figure 3). There were discernible differences in the PCA1 scores between 2 groups; patients with mutations in *DHDDS* and *FAM161A* exhibited lower PCA1 scores, indicative of a more severe retinal disease compared to patients with mutations in *KIZ* and *MAK*. EOG was performed in 6 patients, showing a reduced Arden ratio to values that were lower than those expected by the amplitudes of the ffERG in five out of the six cases (Figure S3).

### 3.2. Characterization of the Expression Pattern of KIZ in Patient-Derived Skin Fibroblasts

**Figure 4:**
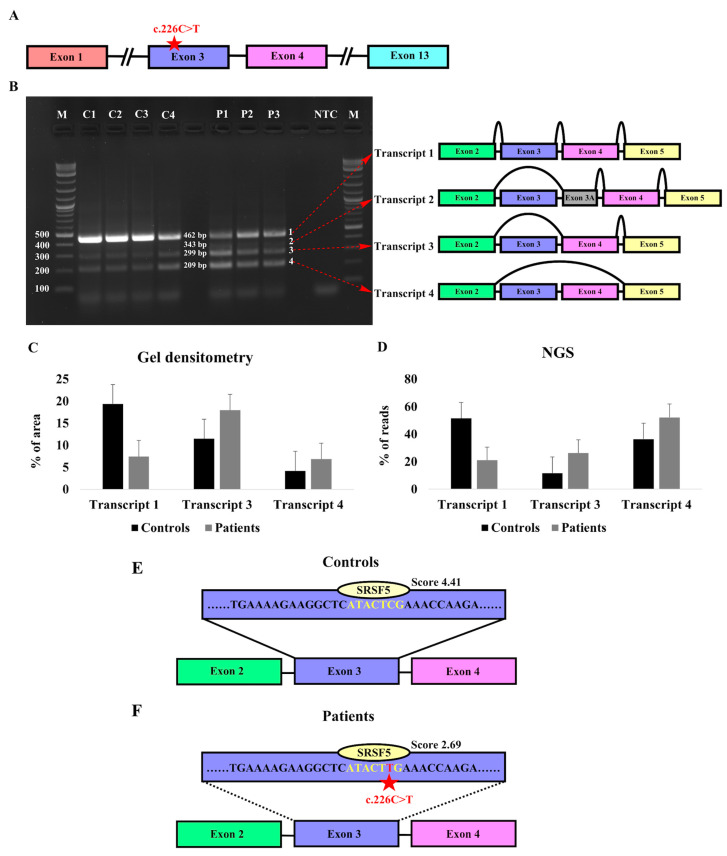
Expression of *KIZ* in human fibroblast cells. (**A**) Schematic structure of the human *KIZ* gene indicating exons that are relevant for the current analysis. The location of the c.226C>T mutation is highlighted. (**B**) RT-PCR agarose gel analysis of primary fibroblast cells from four controls (C1–C4) and three *KIZ* patients (P1-P3). DNA marker is denoted by “M” and negative control by NTC (no template control). Following NGS and Sanger sequencing of the PCR products, the identity of each fragment was determined as schematically depicted on the right. (**C**,**D**) Gel densitometry (**C**) and NGS-based analysis (**D**) of transcripts 1, 3, and 4. Although Transcript 2 was evident in both Sanger and NGS analyses, its expression level was extremely low in all samples and therefore it was excluded from this analysis. (**E**,**F**) Exonic sequence enhancer (ESE) analysis of WT (**E**) and the c.226C>T mutant allele (**F**) of *KIZ* Exon 3. The binding site and score of the SRSF5 protein are shown above the sequence and its putative binding site is shown in yellow. The sequence information of each PCR product can be found in Supplementary Materials.

The *KIZ* gene generates one major transcript encompassing all the coding exons, alongside six minor transcripts primarily characterized by the exclusion of specific exons (Table S5). RT-PCR analysis of RNA isolated from primary fibroblast cell cultures of four control subjects and three patients homozygous for *KIZ*-c.226C>T revealed the presence of four different products (Figure 4A,B). Aiming to identify the corresponding *KIZ* transcripts, we performed both Sanger sequencing and NGS-based sequencing that revealed the following: Transcript 1—the full-length canonical transcript that includes all exons that are flanked by the designed set of primer (exons 2–5), Transcript 2—skipping of exon 3 and inclusion of an alternative exon within intron 3, Transcript 3—skipping of exon 3, and Transcript 4—skipping of both exons 3 and 4. We subsequently used gel densitometry analysis as well as NGS analysis to measure the intensity of each band and the coverage of each transcript as a measure of the expression level (Figure 4C,D). Surprisingly, both analyses revealed lower expression of Transcript 1 in patients compared to controls. On the other hand, a higher expression level was evident for transcripts 3 and 4 (Table S5 and Figure 4C,D). 

Hence, pronounced skipping of exon 3 which carries the pathogenic nonsense variant was evident in patients compared to healthy controls. This observation indicates that the variant may disrupt the normal splicing mechanism. It should be noted here that transcripts 2–4, in which exon 3 is skipped, are out of frame. 

### 3.3. Identification of Exonic Splicing Enhancer (ESE) Sequences in KIZ-Exon 3

ESEs are discrete purine-rich sequences of 6–8 bps located within an exon. These short sequences facilitate exon definition by assisting in the recruitment of splicing factors to the adjacent intron. Changes in the ESE sequence due to mutations may affect the splicing mechanism [30]. We hypothesized that the *KIZ* nonsense variant might have disrupted a potential ESE leading to pronounced exon skipping in patients. 

The analysis of exon 3 sequence using ESEfinder revealed a 7 bp sequence that is recognized by Serine/Arginine-rich Splicing Factor 5 (SRSF5) and includes the c.226th position (Figure 4E,F), with a higher score for the control sequence (ATACTCG-4.411) compared to the mutant sequence (ATACTTG-2.694). The data indicates that the nonsense variant in *KIZ* might affect this ESE sequence, resulting in the pronounced skipping of exon 3, as identified in primary fibroblasts of patients.

### 3.4. Characterization of KIZ Expression in Normal Mice and Sheep Retina

Aiming to characterize the expression of *KIZ* in the retina, we performed RT-PCR analysis on RNA isolated from normal mice (n = 6) and sheep retina (n = 1). We observed only two different transcripts: Transcript 1 corresponds to the full-length canonical transcript and Transcript 2 in which exon 4 is skipped (Figure S4A). NGS analysis revealed a similar expression level of both transcripts (Figure S4B,C). In addition, a small proportion of transcripts (only 0.10%) in which exon 3 is skipped was observed in the mouse retina by NGS (Figure S4D). Therefore, the expression pattern obtained in the mouse and sheep retina is very different from the one obtained in human fibroblast cells.

### 3.5. Analysis of Cilia Generation and Length in Controls and Patients

To examine the functional effect of the *KIZ*-c.226C>T mutation on the cilia, we immunostained the primary fibroblast cells from one control subject and two patients targeting pericentrin and acetylated-α-tubulin. We found that 97.4 ± 3.6% and 96.9 ± 0.9% of cells (n = 100 in each sample) were ciliated in controls and patients, respectively. In addition, the mean ciliary length was measured to be 4.6 ± 0.9 µm and 4.3 ± 0.1 µm in control and patients, respectively (Figure S5). No significant difference was evident in these parameters.

## 4. Discussion

*KIZ* mutations are an uncommon cause of IRDs on a global scale, but they are notably more prevalent among individuals of Ashkenazi Jewish descent, with a carrier rate of 1 in 79 individuals for the p.R76* mutation according to the gnomAD database [31]. This prevalence accounts for the occurrence of the disease in non-consanguineous families. In a previous study, we estimated that 4090 individuals worldwide harbour biallelic *KIZ* mutations, with the majority attributable to the p.R76* variant that is particularly prevalent among individuals of Latino descent [32]. With the inclusion of the current study, a total of 51 biallelic *KIZ* cases associated with ARRP have been reported in the scientific literature, the majority of whom are (31 cases) residing in Israel. Therefore, the current study represents, to the best of our knowledge, the largest cohort of *KIZ*-related ARRP cases reported to date.

All subjects with biallelic *KIZ* mutations were diagnosed with RP but displayed clinical variability in the age of onset and disease progression, similar to the variability reported for other ARRP genes [28,29]. Compared to RP associated with *DHDDS* or *MAK* mutations, patients with *KIZ*-related IRD presented with milder clinical features, although early-onset severe RP was noted in previous studies [22]. The retinal manifestations of *KIZ* patients are often subtle and may be missed on routine clinical examination; hence, it should be noted that all patients exhibited retinal changes that were readily identifiable on fundus autofluorescence. 

Our data suggest that individuals with RP exhibiting either mild or typical retinal abnormalities along with visual field defects displayed a pattern of rod dysfunction exceeding cone dysfunction on a ffERG. This characteristic feature was accompanied by an EOG Arden ratio below the expected level. Hence, patients who belong to Ashkenazi or North-African Jewish ancestry may potentially carry biallelic *KIZ* mutation(s) as the underlying cause of their disease. 

The genetic analysis conducted in this study revealed two unexpected findings. First, we identified four transcripts generated by *KIZ* in WT primary fibroblast cells, three of which are out-of-frame due to exon skipping. In one of these transcripts, exon 3, harbouring the c.226C>T nonsense variant, is skipped. Interestingly, the skipping of exon 3 was found to be enhanced in homozygous patients. This led us to hypothesize not only that c.226C>T introduces a nonsense mutation but might also affect a potential ESE sequence that is recognized by a SRSF. We therefore predict that the c.226C>T variant acts in a dual mutational mechanism, causing the extra skipping of exon 3 (creating a frameshift) and introducing a PTC in a normally spliced transcript. Variants that affect ESEs are difficult to identify [33,34] and only one such variant, c.624G>A in *BEST1*, that is predicted to be a silent variant, has been reported thus far [35]. The effect of this variant on ESEs was studied by sequence analysis and the effect on splicing was investigated using a splicing assay. Since *KIZ* is expressed in fibroblasts, we examined the natural expression pattern of *KIZ* in WT and mutant primary cells. Such dual mechanisms of mutations might have important implications for developing mutation-specific therapeutic modalities. Translation readthrough is currently being studied and developed as a potential therapy for nonsense mutations that cause a variety of inherited diseases, either by readthrough drugs such as ataluren [36,37,38] or by suppressor tRNAs [39,40]. Both methods rely on a substantial number of available transcripts; however, two mechanisms might limit this amount—the nonsense mediated mRNA decay (NMD) that degrades transcripts bearing a PTC [41,42] and the ESE-affecting nonsense mutations, such as *KIZ*-c.226C>T, leading to aberrant splicing. While NMD does not seem to affect *KIZ* transcripts in fibroblasts (and potentially in the retina), the effect of c.226C>T on splicing might limit the number of transcripts for readthrough drugs. RNA editing, which is another mutation-specific therapy that is based on the adenosine deaminases acting on RNA (ADAR) enzyme [43], is usually performed at the pre-mRNA stage [44] and is therefore less affected by NMD; however, editing levels reported thus far for this mutation are relatively low and need to be improved before being considered as a potential therapy [45]. As such, the most suitable therapeutical approach for *KIZ* is currently gene augmentation therapy [46,47,48], which has not yet been developed for this particular gene. 

One of the primary functional assays used to assess the efficacy of readthrough for nonsense mutations in ciliary proteins involves observing the ability of starved mutant fibroblast cells to develop cilia both before and after undergoing readthrough treatment [49]. This approach has been well-established for other retinal proteins that are localized to the photoreceptor cilia, such as USH2A [38], RPGR [50], RP2 [51], and FAM161A [49]. Although KIZ is a known ciliary protein [15], we could not determine any effect of the c.226C>T variant on the ciliary growth of mutant fibroblast cells, in agreement with previous results reported for other *KIZ* mutations [17]. Therefore, ciliary growth cannot serve as a functional assay for assessing KIZ recovery.

A major limitation of the current study, and similarly other studies on retinal diseases, is the type of cells used to understand disease mechanism. Although fibroblast cells that were generated from patient skin biopsies are the most accessible biological material that could be retrieved to understand the pathogenesis of the studied mutation, it does not mimic a precise retinal microenvironment. In addition, a previous study also reported that fibroblasts from patients carrying such truncating mutations is not a suitable system to understand the biology behind the molecular defects in this gene [17] such as expression, protein localization, and functional analyses. Retinal organoids generated from patient-derived iPSCs is likely to be a more suitable model and a reliable alternative to understand the mutation mechanism and to carry out other functional analyses. Similar information might also be obtained from either knockout or knockin mouse models of *KIZ* that are currently unavailable. In addition to the above, our study requires molecular and functional assays to confirm the effect of the mutation on an ESE sequence.

In conclusion, we present the largest cohort of biallelic *KIZ* cases reported to date. The 31 individuals we recruited were all diagnosed with ARRP of varying severity, which on average was slightly greater compared to cases associated with *MAK* mutations, but milder than the phenotype observed in cases linked to *DHDDS* and *FAM161A* mutations. The predominantly identified mutation is c.226C>T that acts as a nonsense mutation as well as an ESE-affecting mutation. 

## Figures and Tables

**Table 1:** Detailed information regarding the 30 identified families with biallelic *KIZ* mutations.

Family Number	Origin	Consanguinity	Mutation 1	Mutation 2	No. Affected Recruited	No. Affected-Not Recruited
MOL0289	Turkish Jew	No	c.226C>T, p.R76*	c.226C>T, p.R76*	1	0
MOL0336	NAJ	2:3	c.226C>T, p.R76*	c.226C>T, p.R76*	1	0
MOL0445	NAJ	No	c.226C>T, p.R76*	c.226C>T, p.R76*	1	0
MOL0588	ASH	No	c.226C>T, p.R76*	c.226C>T, p.R76*	1	1
MOL0610	ASH	3:3	c.226C>T, p.R76*	c.226C>T, p.R76*	1	0
MOL0845	ASH	No	c.226C>T, p.R76*	c.226C>T, p.R76*	1	0
MOL1015	ASH/Turkish Jew	No	c.226C>T, p.R76*	c.226C>T, p.R76*	1	0
MOL1156	ASH	No	c.226C>T, p.R76*	c.226C>T, p.R76*	1	0
MOL1236	AM	2:2	c.247C>T, p.R83*	c.247C>T, p.R83*	1	0
MOL1329	ASH	No	c.226C>T, p.R76*	c.226C>T, p.R76*	1	0
MOL1440	ASH	No	c.226C>T, p.R76*	c.226C>T, p.R76*	2	0
MOL1523	ASH	No	c.226C>T, p.R76*	c.226C>T, p.R76*	1	0
MOL1605	ASH	No	c.226C>T, p.R76*	c.226C>T, p.R76*	1	1
MOL1621	ASH/Iraqi Jew	No	c.226C>T, p.R76*	c.3G>A, p.M1?	1	1
MOL1663	ASH	No	c.226C>T, p.R76*	c.226C>T, p.R76*	1	0
MOL1684	ASH	3:3	c.226C>T, p.R76*	c.226C>T, p.R76*	1	0
MOL1689	ASH	No	c.226C>T, p.R76*	c.226C>T, p.R76*	1	0
MOL1720	ASH	No	c.226C>T, p.R76*	c.226C>T, p.R76*	1	0
MOL1819	NAJ	No	c.226C>T, p.R76*	c.3G>A, p.M1?	1	0
MOL2026	NAJ	No	c.226C>T, p.R76*	c.226C>T, p.R76*	1	0
TB240	ASH	No	c.226C>T, p.R76*	c.226C>T, p.R76*	1	0
TB244	ASH	No	c.226C>T, p.R76*	c.226C>T, p.R76*	1	0
TB338	ASH	No	c.226C>T, p.R76*	c.226C>T, p.R76*	1	0
TB675	ASH	No	c.226C>T, p.R76*	c.226C>T, p.R76*	1	0
TB711	ASH	No	c.226C>T, p.R76*	c.226C>T, p.R76*	1	0
TB736	ASH	No	c.226C>T, p.R76*	c.226C>T, p.R76*	1	0
TB928	ASH	No	c.226C>T, p.R76*	c.226C>T, p.R76*	1	0
TB980	NAJ	No	c.226C>T, p.R76*	c.226C>T, p.R76*	1	0
TB1044	ASH	No	c.226C>T, p.R76*	c.226C>T, p.R76*	1	0
TB1212	ASH	No	c.226C>T, p.R76*	c.226C>T, p.R76*	1	0

NAJ—North-African Jews, ASH—Ashkenazi Jews, AM—Arab Muslim.

## Data Availability

The original contributions presented in the study are included in the article/Supplementary Material, further inquiries can be directed to the corresponding author.

## Supplementary Materials

The following supporting information can be downloaded at: https://www.mdpi.com/article/10.3390/genes15060804/s1, Figure S1: Pedigrees of *KIZ*-related families; Figure S2: Comparison of clinical parameters by two-sided *t*-test; Figure S3: EOG versus ERG data; Figure S4: RT-PCR analysis of normal mice and sheep retina; Figure S5: Ciliary analysis of c.226C>T fibroblasts; Table S1: Reported mutations in *KIZ*; Table S2: Clinical information of patients will biallelic *KIZ* pathogenic variants; Table S3: The seven principal components sample variances obtained in this study; Table S4: The variable coefficient in each of the seven principal component; Table S5: *KIZ* transcript information.

## References

[1] Schneider N., Sundaresan Y., Gopalakrishnan P., Beryozkin A., Hanany M., Levanon E.Y., Banin E., Ben-Aroya S., Sharon D. (2021). Inherited Retinal Diseases: Linking Genes, Disease-Causing Variants, and Relevant Therapeutic Modalities. Prog. Retin. Eye Res..

[2] den Hollander A.I., Roepman R., Koenekoop R.K., Cremers F.P. (2008). Leber Congenital Amaurosis: Genes, Proteins and Disease Mechanisms. Prog. Retin. Eye Res..

[3] Verbakel S.K., van Huet R.A.C., Boon C.J.F., den Hollander A.I., Collin R.W.J., Klaver C.C.W., Hoyng C.B., Roepman R., Klevering B.J. (2018). Non-Syndromic Retinitis Pigmentosa. Prog. Retin. Eye Res..

[4] Ali M.U., Rahman M.S.U., Cao J., Yuan P.X. (2017). Genetic Characterization and Disease Mechanism of Retinitis Pigmentosa; Current Scenario. 3 Biotech.

[5] Hartong D.T., Berson E.L., Dryja T.P. (2006). Retinitis Pigmentosa. Lancet.

[6] Berson E.L. (1993). Retinitis Pigmentosa. The Friedenwald Lecture. Investig. Ophthalmol. Vis. Sci..

[7] Parmar U.P.S., Surico P.L., Singh R.B., Romano F., Salati C., Spadea L., Musa M., Gagliano C., Mori T., Zeppieri M. (2024). Artificial Intelligence (AI) for Early Diagnosis of Retinal Diseases. Medicina.

[8] Georgiou M., Robson A.G., Fujinami K., de Guimarães T.A.C., Fujinami-Yokokawa Y., Daich Varela M., Pontikos N., Kalitzeos A., Mahroo O.A., Webster A.R. (2024). Phenotyping and Genotyping Inherited Retinal Diseases: Molecular Genetics, Clinical and Imaging Features, and Therapeutics of Macular Dystrophies, Cone and Cone-Rod Dystrophies, Rod-Cone Dystrophies, Leber Congenital Amaurosis, and Cone Dysfunction Syndromes. Prog. Retin. Eye Res..

[9] Ferrari S., Di Iorio E., Barbaro V., Ponzin D., Sorrentino F.S., Parmeggiani F. (2011). Retinitis Pigmentosa: Genes and Disease Mechanisms. Curr. Genom..

[10] Ayuso C., Millan J.M. (2010). Retinitis Pigmentosa and Allied Conditions Today: A Paradigm of Translational Research. Genome Med..

[11] Chen H.Y., Welby E., Li T., Swaroop A. (2019). Retinal Disease in Ciliopathies: Recent Advances with a Focus on Stem Cell-Based Therapies. Transl. Sci. Rare Dis..

[12] Tatour Y., Ben-Yosef T. (2020). Syndromic Inherited Retinal Diseases: Genetic, Clinical and Diagnostic Aspects. Diagnostics.

[13] Chandra B., Tung M.L., Hsu Y., Scheetz T., Sheffield V.C. (2022). Retinal Ciliopathies through the Lens of Bardet-Biedl Syndrome: Past, Present and Future. Prog. Retin. Eye Res..

[14] Chen H.Y., Kelley R.A., Li T., Swaroop A. (2021). Primary Cilia Biogenesis and Associated Retinal Ciliopathies. Semin. Cell Dev. Biol..

[15] El Shamieh S., Neuillé M., Terray A., Orhan E., Condroyer C., Démontant V., Michiels C., Antonio A., Boyard F., Lancelot M.-E. (2014). Whole-Exome Sequencing Identifies KIZ as a Ciliary Gene Associated with Autosomal-Recessive Rod-Cone Dystrophy. Am. J. Hum. Genet..

[16] Gustafson K., Duncan J.L., Biswas P., Soto-Hermida A., Matsui H., Jakubosky D., Suk J., Telenti A., Frazer K.A., Ayyagari R. (2017). Whole Genome Sequencing Revealed Mutations in Two Independent Genes as the Underlying Cause of Retinal Degeneration in an Ashkenazi Jewish Pedigree. Genes.

[17] El Shamieh S., Méjécase C., Bertelli M., Terray A., Michiels C., Condroyer C., Fouquet S., Sadoun M., Clérin E., Liu B. (2017). Further Insights into the Ciliary Gene and Protein KIZ and Its Murine Ortholog PLK1S1 Mutated in Rod-Cone Dystrophy. Genes.

[18] Méjécase C., Kozak I., Moosajee M. (2020). The Genetic Landscape of Inherited Eye Disorders in 74 Consecutive Families from the United Arab Emirates. Am. J. Med. Genet. C Semin. Med. Genet..

[19] Jauregui R., Chan L., Oh J.K., Cho A., Sparrow J.R., Tsang S.H. (2020). Disease Asymmetry and Hyperautofluorescent Ring Shape in Retinitis Pigmentosa Patients. Sci. Rep..

[20] Sharon D., Ben-Yosef T., Goldenberg-Cohen N., Pras E., Gradstein L., Soudry S., Mezer E., Zur D., Abbasi A.H., Zeitz C. (2019). A Nation-wide Genetic Analysis of Inherited Retinal Diseases in Israel as Assessed by the Israeli Inherited Retinal Disease Consortium (IIRDC). Hum. Mutat..

[21] Weisschuh N., Obermaier C.D., Battke F., Bernd A., Kuehlewein L., Nasser F., Zobor D., Zrenner E., Weber E., Wissinger B. (2020). Genetic Architecture of Inherited Retinal Degeneration in Germany: A Large Cohort Study from a Single Diagnostic Center over a 9-Year Period. Hum. Mutat..

[22] Lin Y., Xu C.L., Breazzano M.P., Tanaka A.J., Ryu J., Levi S.R., Yao K., Sparrow J.R., Tsang S.H. (2020). Progressive RPE Atrophy and Photoreceptor Death in KIZ-Associated Autosomal Recessive Retinitis Pigmentosa. Ophthalmic Genet..

[23] Villafuerte-de la Cruz R.A., Garza-Garza L.A., Garza-Leon M., Rodriguez-De la Torre C., Parra-Bernal C., Vazquez-Camas I., Ramos-Gonzalez D., Rangel-Padilla A., Espino Barros-Palau A., Nava-García J. (2024). Spectrum of Variants Associated with Inherited Retinal Dystrophies in Northeast Mexico. BMC Ophthalmol..

[24] Zhao Y., Coussa R.G., DeBenedictis M.J.M., Traboulsi E.I. (2019). Retinal Dystrophy Associated with a Kizuna (KIZ) Mutation and a Predominantly Macular Phenotype. Ophthalmic Genet..

[25] Oshimori N., Ohsugi M., Yamamoto T. (2006). The Plk1 Target Kizuna Stabilizes Mitotic Centrosomes to Ensure Spindle Bipolarity. Nat. Cell Biol..

[26] Thompson D.A., Bach M., McAnany J.J., Šuštar Habjan M., Viswanathan S., Robson A.G. (2024). ISCEV standard for clinical pattern electroretinography (2024 update). Doc. Ophthalmol..

[27] Cartegni L., Wang J., Zhu Z., Zhang M.Q., Krainer A.R. (2003). ESEfinder: A Web Resource to Identify Exonic Splicing Enhancers. Nucleic Acids Res..

[28] Kimchi A., Khateb S., Wen R., Guan Z., Obolensky A., Beryozkin A., Kurtzman S., Blumenfeld A., Pras E., Jacobson S.G. (2018). Nonsyndromic Retinitis Pigmentosa in the Ashkenazi Jewish Population. Genetic and Clinical Aspects. Ophthalmology.

[29] Beryozkin A., Khateb S., Idrobo-Robalino C., Khan M., Cremers F., Obolensky A., Hanany M., Mezer E., Chowers I., Newman H. (2020). Unique Combination of Clinical Features in a Large Cohort of 100 Patients with Retinitis Pigmentosa Caused by FAM161A Mutations. Sci. Rep..

[30] Sundaresan Y., Banin E., Sharon D. (2023). Exonic Variants That Affect Splicing—An Opportunity for “Hidden” Mutations Causing Inherited Retinal Diseases. Adv. Exp. Med. Biol..

[31] Chen S., Francioli L.C., Goodrich J.K., Collins R.L., Kanai M., Wang Q., Alföldi J., Watts N.A., Vittal C., Gauthier L.D. (2024). A Genomic Mutational Constraint Map Using Variation in 76,156 Human Genomes. Nature.

[32] Hanany M., Rivolta C., Sharon D. (2020). Worldwide Carrier Frequency and Genetic Prevalence of Autosomal Recessive Inherited Retinal Diseases. Proc. Natl. Acad. Sci. USA.

[33] Liu H.X., Zhang M., Krainer A.R. (1998). Identification of Functional Exonic Splicing Enhancer Motifs Recognized by Individual SR Proteins. Genes. Dev..

[34] Xu D.Q., Mattox W. (2006). Identification of a Splicing Enhancer in MLH1 Using COMPARE, a New Assay for Determination of Relative RNA Splicing Efficiencies. Hum. Mol. Genet..

[35] Kramer F., Mohr N., Kellner U., Rudolph G., Weber B.H. (2003). Ten Novel Mutations in VMD2 Associated with Best Macular Dystrophy (BMD). Hum. Mutat..

[36] Finkel R.S., Flanigan K.M., Wong B., Bonnemann C., Sampson J., Sweeney H.L., Reha A., Northcutt V.J., Elfring G., Barth J. (2013). Phase 2a Study of Ataluren-Mediated Dystrophin Production in Patients with Nonsense Mutation Duchenne Muscular Dystrophy. PLoS ONE.

[37] Wilschanski M., Miller L.L., Shoseyov D., Blau H., Rivlin J., Aviram M., Cohen M., Armoni S., Yaakov Y., Pugatsch T. (2011). Chronic Ataluren (PTC124) Treatment of Nonsense Mutation Cystic Fibrosis. Eur. Respir. J..

[38] Samanta A., Stingl K., Kohl S., Nagel-Wolfrum K., Ries J., Linnert J. (2019). Ataluren for the Treatment of Usher Syndrome 2A Caused by Nonsense Mutations. Int. J. Mol. Sci..

[39] Wang J., Zhang Y., Mendonca C.A., Yukselen O., Muneeruddin K., Ren L., Liang J., Zhou C., Xie J., Li J. (2022). AAV-Delivered Suppressor TRNA Overcomes a Nonsense Mutation in Mice. Nature.

[40] Albers S., Allen E.C., Bharti N., Davyt M., Joshi D., Perez-Garcia C.G., Santos L., Mukthavaram R., Delgado-Toscano M.A., Molina B. (2023). Engineered TRNAs Suppress Nonsense Mutations in Cells and in Vivo. Nature.

[41] Hentze M.W., Kulozik A.E. (1999). A Perfect Message: RNA Surveillance and Nonsense-Mediated Decay. Cell.

[42] Benslimane N., Loret C., Chazelas P., Favreau F., Faye P.A., Lejeune F., Lia A.S. (2024). Readthrough Activators and Nonsense-Mediated MRNA Decay Inhibitor Molecules: Real Potential in Many Genetic Diseases Harboring Premature Termination Codons. Pharmaceuticals.

[43] Merkle T., Stafforst T. (2021). New Frontiers for Site-Directed RNA Editing: Harnessing Endogenous ADARs. Methods Mol. Biol..

[44] Booth B.J., Nourreddine S., Katrekar D., Savva Y., Bose D., Long T.J., Huss D.J., Mali P. (2023). RNA Editing: Expanding the Potential of RNA Therapeutics. Mol. Ther..

[45] Schneider N., Steinberg R., Ben-David A., Valensi J., David-Kadoch G., Rosenwasser Z., Banin E., Levanon E.Y., Sharon D., Ben-Aroya S. (2024). A Pipeline for Identifying Guide RNA Sequences That Promote RNA Editing of Nonsense Mutations That Cause Inherited Retinal Diseases. Mol. Ther. Nucleic Acids.

[46] Michalakis S., Gerhardt M., Rudolph G., Priglinger S., Priglinger C. (2021). Gene Therapy for Inherited Retinal Disorders: Update on Clinical Trials. Klin. Monbl Augenheilkd..

[47] Botto C., Rucli M., Tekinsoy M.D., Pulman J., Sahel J.-A., Dalkara D. (2022). Early and Late Stage Gene Therapy Interventions for Inherited Retinal Degenerations. Prog. Retin. Eye Res..

[48] Brar A.S., Parameswarappa D.C., Takkar B., Narayanan R., Jalali S., Mandal S., Fujinami K., Padhy S.K. (2024). Gene Therapy for Inherited Retinal Diseases: From Laboratory Bench to Patient Bedside and Beyond. Ophthalmol. Ther..

[49] Beryozkin A., Samanta A., Gopalakrishnan P., Khateb S., Banin E., Sharon D., Nagel-Wolfrum K. (2022). Translational Read-Through Drugs (TRIDs) Are Able to Restore Protein Expression and Ciliogenesis in Fibroblasts of Patients with Retinitis Pigmentosa Caused by a Premature Termination Codon in FAM161A. Int. J. Mol. Sci..

[50] Vössing C., Owczarek-Lipska M., Nagel-Wolfrum K., Reiff C., Jüschke C., Neidhardt J. (2020). Translational Read-through Therapy of Rpgr Nonsense Mutations. Int. J. Mol. Sci..

[51] Schwarz N., Carr A.J., Lane A., Moeller F., Chen L.L., Aguilà M., Nommiste B., Muthiah M.N., Kanuga N., Wolfrum U. (2015). Translational Read-through of the RP2 Arg120stop Mutation in Patient IPSC-Derived Retinal Pigment Epithelium Cells. Hum. Mol. Genet..

