# Supplementary material for: Genetic and Clinical Analyses of the KIZ-c.226C>T Variant Resulting in a Dual Mutational Mechanism
Source: Genes (Basel). 2024 Jun 18;15(6):804. doi: 10.3390/genes15060804 (PMC11202946; doi:10.3390/genes15060804)
Supplement: Supplementary file 1 [file genes-15-00804-s001.zip › genes-3051641-supplementary.pdf]

Supplemental Information File

Genetic and Clinical Analyses of the *KIZ*-c.226C>T Variant Resulting in a Dual Mutational Mechanism

Yogapriya Sundaresan [1,†], Antonio Rivera [1,†], Alexey Obolensky [1], Prakadeeswari Gopalakrishnan [1], Hanit Ohayon Hadad [1], Aya Shemesh [1], Samer Khateb [1], Maya Ross [2], Ron Ofri [2], Sharon Durst [1], Hadas Newman [3], Rina Leibu [4], Shiri Soudry [5], Dinah Zur [3], Tamar Ben-Yosef [6], Eyal Banin [1,‡] and Dror Sharon [1,*,‡]

[1] Department of Ophthalmology, Hadassah Medical Center, Faculty of Medicine, Hebrew University of Jerusalem, Jerusalem 91120, Israel; yogapriya.sundar@mail.huji.ac.il (Y.S.); antonioriveravasq@gmail.com (A.R.); prakadee.gopalakrish@mail.huji.ac.il (P.G.); banine@mail.huji.ac.il (E.B.)
[2] Koret School of Veterinary Medicine, The Hebrew University of Jerusalem, Rehovot 76100, Israel
[3] Ophthalmology Division, Tel Aviv Sourasky Medical Center, Affiliated to Faculty of Medical & Health Sciences, Tel Aviv University, Tel Aviv 69978, Israel
[4] Department of Ophthalmology, Rambam Health Care Center, Haifa 31096, Israel
[5] Department of Ophthalmology, Rabin Medical Center, Petah Tikva 49100, Israel
[6] The Ruth & Bruce Rappaport Faculty of Medicine, Technion-Israel Institute of Technology, Haifa 31096, Israel
* Correspondence: dror.sharon1@mail.huji.ac.il; Tel.: +972-2-6777112; Fax: +972-2-6448917
† These authors contributed equally to this work.
‡ Shared last authors.

Supplemental Clinical Note:

Case series:

MOL0289-3 is an isolate case from a non-consanguineous Turkish Jewish family. In her late teens she started experiencing difficulty in night vision and was presented with mild decrease in visual acuity (VA) until her last examination at the age of 33 years. She had a mild posterior subcapsular cataract (PSC) in both eyes, which required no surgery. VA at the age of 25 years was 0.63 in RE and 0.6 in LE, which decreased to 0.4 in both eyes by the age of 33 years. Goldman visual field at the age of 23 years was relatively preserved but by the age of 26 years there was a 10 degree constriction. Funduscopic findings showed mild RPE mottling with atrophy near the arcades, with a few scattered spots of bone spicule like pigmentation, attenuated vessels, with the temporal arcades reaching only the mid-periphery and thinning of the nasal arcades. On fundus autofluorescence (FAF), more hypoautofluorescent spots surrounding the arcades were seen and a hyperautofluorescent ring around the fovea was evident. Optical coherence tomography (OCT) showed a very mild epiretinal membrane (ERM) with loss of the photoreceptor layer, and the small island was preserved in the fovea.

MOL0336-1 is of a Moroccan Jewish ancestry with consanguinity. The father, the grandfather and an uncle were apparently affected. The father was blind at an old age and the patient started complaining of night blindness at the age of 35 years. However, the patient had a good VA at this point of time with a 0.5 in RE and 0.63 in LE. Refraction was insignificant, and there were no signs of cataract. Full-field ERG (ffERG) showed a rod cone pattern of dysfunction, with recordable amplitudes. The

fundus findings described a classical RP, with bone spicule like pigmentation in the mid periphery forming a ring and retinal attenuation.

MOL0445-1 is of a Moroccan Jewish origin with no reported consanguinity and is the only affected individual in the family. The patient complained on night blindness since childhood and was diagnosed with RP at the age of 18 years. At the age of 23 years , VA was well preserved with 1.0 in both eyes, refraction was insignificant and no cataract was reported. At the age of 29 years, there was a mild reduction in VA in both RE and LE (0.63 and 0.8). Minimal PSC was evident. The fundus findings (Figure 2) revealed a bone spicule like pigmentation in the mid periphery, RPE mottling, attenuation of vessels and pallor of the optic disc and WWP in the far periphery. FAF showed a hyperautofluorescent ring around the fovea with a multiple hypoautofluorescent spots along the arcades forming a ring in the mid periphery. OCT showed a very mild ERM, thinning of all the retinal layers with loss of the photoreceptor layer, and an island of ellipsoid zone (EZ) in the fovea.

MOL0610-1 is of an Ashkenazi Jewish origin from the former Soviet Union. The parents were distant cousins and the patient was the only affected individual in the family. At the age of 38 years, VA was well preserved in both the RE (1.0) and LE (0.8) exhibiting no signs of cataract. Visual fields at the age of 39 years were restricted to 20 degrees. The fundus findings described areas of atrophy with pigmentation along the inferior arcade in both the eyes with no cystoid macular edema (CME). OCT showed loss of the photoreceptor layer that was only preserved in the fovea.

MOL0845-1 is of an Ashkenazi Jewish origin and is the only case in the family. The patient was diagnosed with RP at the age of 23. By 27 years of age, the VA was 0.3

in the RE and 0.5 in the LE with no signs of cataract, which progressed up to HM in both eyes by the age of 57 years. In addition, severe PSC was evident. Cataract surgery was performed in both eyes, but no objective improvement in VA was seen. Visual fields were restricted to less than 10 degrees by the age of 34 years. The fundus findings at the age of 65 years (Figure 2) showed advanced RP with bone spicule like pigmentation dispersed around the retina, atrophy around the arcades, pallor of the optic disc and retinal vessel attenuation with ghost vessels. The LE had a very blurry picture due to severe posterior capsule opacification and phimosis of the capsule. On FAF, patches of hypoautofluorescence surrounding the arcades, the far periphery was spared and a hyperautofluorescent ring in the macula was seen. OCT showed an ERM with mild traction of the fovea. However, no intraretinal fluid or loss of the integrity of the layers was seen. Additionally, marked thinning of the ONL and an a very small island of EZ in the fovea was evident.

MOL1015-1 is of a mixed family background. The father is an Ashkenazi Jew from Poland and the mother is from Greece / The Netherlands. The patient is the only affected individual in the family. The patient was diagnosed with amblyopia in the LE at a young age, and did not exhibit any visual impairment until the age of 40 years. The patient started experiencing defects in the visual field only at the age of 40 years. During the first examination at age of 63 years, the visual acuity was 0.05 in RE and 0.1 in LE, with mild nuclear and PSC in both eyes. At the age of 63 years, 120 point visual field screening revealed a completely black visual field. Fundus findings revealed BE large optic disc cupping, highly attenuated retinal vessels, and the presence of only ghost vessels in the periphery. A bone spicule like pigmentation forming a concentric ring was evident in the mid periphery. Macular atrophy was noted in both eyes, however was predominant in the right.

MOL1440-2 is of an Ashkenazi Jewish origin and has one brother (MOL1440-1) with the same disease and genetic mutation. The patient was presented to our clinic at the age of 43 years. The patient was diagnosed with amblyopia in the LE since childhood, started experiencing decreased VA in her early 20's, and was diagnosed with RP at age of 42. In her first visit, visual acuity was 0.63 in RE and 0.02 in LE, with a mild hyperopia (+3) in both eyes. Visual fields of the 24-2 type were completely black at this stage. Fundus findings (Figure 2) showed atrophy surrounding the arcades with mild pigmentary changes. On FAF, there were multiple patches of hypoautofluorescence scattered along the arcades. OCT revealed thinning of the ONL, mild ERM, and an island of EZ in the fovea.

MOL1523-1 is of an Ashkenazi Jewish origin from England with no reported consanguinity and is the only affected individual in the family. The patient was being treated for normal tension glaucoma due to progressive visual field defects in England, and after an incidental finding of pigmentary changes in the retina, the diagnosis was changed to retinal degeneration. VA was very good up to the last examination at the age of 66 years, with a 0.5 in RE and 1.0 in LE. Mild nuclear cataract with no signs of PSC was observed. In addition the patient had high hypermetropia (RE +8 and LE +5). Visual fields were well preserved at the age of 63 years. The funduscopic findings at the age of 63 years (Figure 2) showed very mild RPE mottling with atrophy near the arcades and the rest of the fundus appeared normal. On FAF there is a hyperautofluorescent ring around the fovea with a few hypoautofluorescent spots confined to the macular area. On OCT, thinning of all the retinal layers with loss of the photoreceptor layer was observed and the small Island was preserved in the fovea.

MOL1605-1 is the index case from a non-consanguineous Ashkenazi Jewish family. She has one brother diagnosed with RP, and nine sisters and three brothers who are unaffected. She was first referred to our center because of an elevated pigmented lesion in the left eye, which was diagnosed as Choroidal nevus vs Melanocytoma. During this visit, she complained on night blindness and due to the family history, she was sent for further evaluation. Her VA was very variable in all of her examinations, from 0.4 to 0.8 in the RE and from 0.1 to 0.6 in her LE and this may be due to the cystic macular changes in the retina found on OCT. Until her last examination at the age of 19 years, there were no signs of cataract. Visual fields were relatively well preserved at age of 14 years, but showed multiple scotomas in her last Octopus visual field at the age of 17 years. Funduscopic findings (Figure 2) showed mild RPE mottling with atrophy near the arcades, with a few scattered spots of bone spicule like pigmentation. Elevated pigmented lesion peripapillary in the LE was noted, which fits the diagnosis of Melanocytoma, and another two pigmented spots in the inferior arcade supports the diagnosis of choroidal nevi. On FAF (Figure 2), a hyperautofluorescent ring around the fovea with a few hypoautofluorescent spots scattered in the mid periphery was seen. Another hyperautofluorescent ring was noted in the mid periphery surrounding the macula. On OCT, there was a very mild ERM with loss of the photoreceptor layer, small Island was preserved in the fovea and CME was present in both eyes.

MOL1621-1 is of a mixed family background where the father is Iraqi Jew and mother is an Ashkenazi Jew from Poland. This indicates that the parents were not related. The patient has two sisters diagnosed with no eye disease. The patient carries a compound heterozygous mutation and started experiencing progressive night blindness and visual field defects from the 3rd decade of life. Further, the

patient underwent LASIK refractive surgery due to mild myopia. The first eye examination in our center was carried at the age of 42 years where the VA was measured to be 0.4 in RE and 0.25 in LE. Visual field test at the age of 36 years reported tunnel vision with preservation of the temporal peripheral field and ERG was reported to be normal. Description of the fundus findings showed few pigmentary spots with mild atrophy of the retina and OCT revealed thinning of the photoreceptor layer.

MOL1663-1 is of an Ashkenazi Jewish origin with no reported consanguinity and is the only affected individual in the family. At the age of 45 years, the patient experienced difficulty in night vision and was also presented with constricted visual field. However, good VA was observed in both the eyes (0.63) which was preserved until the last examination (0.5). Severe and moderate PSC was observed in LE and RE respectively. Highly reduced visual fields, constricted to 20 degrees was noticed and this progressed to 10 degrees in the next 3 years. Fundus findings at the age of 64 years (Figure 2) identified a bone spicule like pigmentation in the mid periphery along with RPE atrophy, waxy pallor of the optic disc and attenuation of vessels, including ghost vessels. OCT revealed thinning of all the retinal layers with loss of the photoreceptor layer, preserved only in the fovea.

MOL1684-1 is of an Ashkenazi Jewish origin from Russia with reported consanguinity. The patient started experiencing difficulty in night vision during his early 20's and underwent ffERG at age 30 when the diagnosis of RP was given. The first examination of the patient was carried out in our department when he was 58 years of age. VA was already at the level of RE HM and LE FC 30cm, with a mild PSC in both eyes. At age 56 years, visual fields were completely black. The fundus findings described diffuse retinal atrophy and bone spicules in the mid periphery.

MOL1689-1 is of an Ashkenazi Jewish origin with no reported consanguinity and has three siblings including a non-identical twin with no signs of ocular diseases. He started having difficulty in night vision at 20 years of age. VA was very good until the last examination at the age of 23 years (RE =1.0 and LE =0.6) and there were no signs of cataract. Goldman visual fields were relatively preserved at the age of 21 years. The funduscopic findings at the age of 23 years (Figure 2) showed very mild RPE mottling with atrophy near the arcades, mild attenuation of vessels, and pallor of the optic disc. The rest of the fundus appeared normal. On FAF, a hyperautofluorescent ring surrounding the fovea with another hyperautofluorescent ring in the mid periphery surrounding the macula was noted. On OCT, a very mild ERM with loss of the photoreceptor layer was observed. Small island was preserved in the fovea.

MOL1720-1 is of an Ashkenazi Jewish origin from Poland with no reported consanguinity and is the only affected individual in the family. The patient started complaining on difficulty in night vision, as well as photophobia from the 4th decade of life. At 50 years of age, VA was well preserved having a 0.5 in both eyes. Mild nuclear cataract with no signs of PSC was observed. The funduscopic findings showed very mild RPE mottling with atrophy near the arcades. On FAF there is a hyperautofluorescent ring around the fovea with a few hypoautofluorescent spots scattered in the mid periphery. Another hyperautofluorescent ring was found in the mid periphery surrounding the macula. On OCT, loss of the photoreceptor layer was evident and the small Island was preserved in the fovea.

MOL1819-1 is of a mixed family background, where the father is a Spanish Jew and the mother is a Macedonian Jew. This indicated that the parents were not related. The patient harboured compound heterozygous mutations. The maternal grandfather

had a history of night blindness and loss of visual function by 50 years of age. The patient started feeling difficulty at night especially while driving in the 4th decade of life. The patient realized his military service in the air force and underwent eye examinations that only revealed minimal astigmatism. According to the patient, the first sign may have been visual field defects in the 3rd decade. VA was very well preserved by 50 years of age with a 1.0 in RE and 0.9 in LE, and no signs of cataract. Humphrey 24-2 visual fields at this age showed practically a black visual field. The funduscopic findings (Figure 2) showed very mild RPE mottling with atrophy near the arcades, and the rest of the fundus was normal. On FAF, a hyperautofluorescent ring around the fovea with a multiple hypoautofluorescent spots along the arcades was observed and another hyperautofluorescent ring around the macula in the mid periphery was evident. On OCT, loss of the photoreceptor layer, mild ERM, and a small preserved Island in the fovea was evident.

Supplemental Methods

Culturing the fibroblast cells from skin biopsies

KIZ patient-derived fibroblasts homozygous for the nonsense mutation c.226C>T (p.R76*) and fibroblasts from healthy controls, were grown in RPMI-1640 with L-Glutamine (01-100-1A – Biological Industries) containing 15% Fetal calf serum (FCS), 1% penicillin-streptomycin, 1% sodium pyruvate and 1% non – essential amino acids at 37°C with 5% CO_2 cell culture incubator.

Establishment of primary fibroblast cells from tissue explants

Fibroblasts were establish from skin biopsies of four healthy controls and three *KIZ* patients homozygous for c.226C>T using the following protocol. The skin biopsy was taken using a 1.5mm puncher and was immediately transferred to 500µl sterile RPMI complete growth medium in a 60mm petri dish and disintegrated. The tissue sample was incubated in a 37ºc until confluency (by 3 weeks). and to obtain pure fibroblast cultures.

Seeding for ciliogenesis

The patient-derived and wildtype fibroblast cells were seeded in six-well plates containing 15 *mm* diameter coverslips. 1.4 x 10^5 cells per well were seeded for both patient-derived and wildtype controls. To each well 2ml of the complete growth medium (RPMI-15% FCS) was added. After 24 hours of incubation in 37°C with 5% CO_2, the cells were serum-starved by culturing in OPTI-MEM reduced serum medium (Thermo Fisher Scientific, Waltham, MA) for 48 hours. Following serum-starvation, Ataluren (10µg/ml) treatment was carried out in reduced serum media and untreated cells served as controls. After 48 hours of the treatment, the cells grown on the coverslips were taken for downstream immunocytochemistry labelling for cilia and *KIZ*.

Immunocytochemistry and microscopy

The cells were washed with 1X PBS (Phosphate-buffered saline) and fixed using 4% PFA (Paraformaldehyde) diluted in 1X PBS for 10 minutes at room temperature

followed by 3 washes with ice-cold PBS. To achieve permeabilization of the cells, the cells were incubated with 0.25% Triton X-100 diluted in 1X PBS for 10 minutes. Three PBS washes were carried out for 5 minutes each. 1% blocking solution was added to the cells and incubated for 1 hour at room temperature. The blocking solution was removed and primary antibodies [Anti-acetylated-α-tubulin (Sigma, St. Louis, MO; T6793, at a dilution of 1:1000), and anti-pericentrin (Abcam, Cambridge, UK; ab99341, 1:750)] diluted using fresh 1% bovine serum albumin (BSA) were added to the cells and incubated overnight at 4°C. Appropriate secondary antibodies [goat-anti-mouse 594 and goat-anti-rabbit 488 (Abcam, Cambridge, UK) were used at a dilution of 1:250 each, diluted in 1X PBS], after which the cells were washed thrice in 1X PBS and mounted with mounting media containing DAPI (Vector Laboratories, Burlingame, CA). Images of more than 100 consecutive immunostained cells were acquired using fluorescent microscope (Olympus, Tokyo, Japan) from each sample. The DAPI stained nucleus was acquired using UV laser and acetylated-α-tubulin as well as pericentrin was imaged using green and blue laser, respectively. The percentage of the ciliated cells and the length of the cilia was calculated using ImageJ.

Analysis of cilia generation and length

A minimum of 100 fibroblasts were analyzed from each sample to determine the presence of cilia using ImageJ software. Cells stained for both acetylated-α-tubulin and pericentrin were considered as ciliated cells. The percentage of ciliated cells were calculated based on the number of cells possessing the cilia among the total number of cells included in the analysis. The length of the cilia was determined by

measuring the whole cilia (from the basal body to the distal end of the axoneme) using ImageJ.

Supplemental Sequences

Below are the sequences of the various PCR products described in Figure 4:

Transcript 1 (Canonical): 462bp- 395 (Ex2-5) + 67 (adapter)

AAAAGAAGAGATTGGACCTGGAAAAGAAACTTTATGAATATAATCAGTCTGATACATGCA
GAGTTAAGCTGAAATATGTAAAACTAAAGAATTATCTGAAGGAAATATGTGAATCTGAAA
AGAAGGCTCATACTCGAAACCAAGAATATTTAAAGCGATTTGAGCGTGTCCAAGCTCAT
GTTGTACACTTCACCACAAATACAGAGAAGCTTCAAAAACTGAAGCTCGAATATGAGAC
TCAAATTAAGAAGATGCTATGCTCAAAAGATAGCCTGGGACTAAAAGAGGAACTGACAG
ATGAAGACAGAGAAAAGGTTGCAGTGCACGAGGGGATTAACTCAGGAACAGCCATGTC
AAGAGGATTGTATCAACCAGCAACAATCTTTATGGGCCGC

Transcript 2 (Exon 3 deletion + 26 bp exon): 325bp- 232 (Ex2,4,5) + 26 (Ex3a)+ 67bp (adapter)

AAAAGAAGAGATTGGACCTGGAAAAGAAACTTTATGAATATAATCAGTCTGATACATGCA
GAGACCTGGAGAACAGGGATTTGACGCCTCGAATATGAGACTCAAATTAAGAAGATGCT
ATGCTCAAAAGATAGCCTGGGACTAAAAGAGGAACTGACAGATGAAGACAGAGAAAAG
GTTGCAGTGCACGAGGGGATTAACTCAGGAACAGCCATGTCAAGAGGATTGTATCAAC
CAGCAACAATCTTTATGGGCCGC

Transcript 3 (Exon 3 deletion): 299bp- 232 (Ex2,4,5) + 67bp (adapter)

AAAAGAAGAGATTGGACCTGGAAAAGAAACTTTATGAATATAATCAGTCTGATACATGCA
GCTCGAATATGAGACTCAAATTAAGAAGATGCTATGCTCAAAAGATAGCCTGGGACTAA
AAGAGGAACTGACAGATGAAGACAGAGAAAAGGTTGCAGTGCACGAGGGGATTAACTC
AGGAACAGCCATGTCAAGAGGATTGTATCAACCAGCAACAATCTTTATGGGCCGC

Transcript 4 (Exon 3 and 4 deletion): 209bp- 142 (Ex2,5) + 67bp (adapter)

AAAAGAAGAGATTGGACCTGGAAAAGAAACTTTATGAATATAATCAGTCTGATACATGCA
GGTTGCAGTGCACGAGGGGATTAACTCAGGAACAGCCATGTCAAGAGGATTGTATCAA
CCAGCAACAATCTTTATGGGCCGC

**Forward Primer**	AAAAGAAGAGATTGGACCTG	20 bp
**Reverse Primer**	CAACAATCTTTATGGGCCGC	20bp
**Exon 2**	GAAAAGAAACTTTATGAATATAATCAGTCTGATACATGCAG	
**Exon 3**	AGTTAAGCTGAAATATGTAAAACTAAAGAATTATCTGAAGGAAATA<br>TGTGAATCTGAAAAGAAGGCTCATACTCGAAACCAAGAATATTTA<br>AAGCGATTTGAGCGTGTCCAAGCTCATGTTGTACACTTCACCACA<br>AATACAGAGAAGCTTCAAAAACTGAAG	163 bp

**Exon 3A**	AGACCTGGAGAACAGGGATTTGACGC	26 bp
**Exon 4**	CTCGAATATGAGACTCAAATTAAGAAGATGCTATGCTCAAAAGATAGCCTGGGACTAAAAGAGGAACTGACAGATGAAGACAGAGAAAAG	90 bps
**Exon 5**	GTTGCAGTGCACGAGGGGATTAACTCAGGAACAGCCATGTCAAGAGGATTGTATCAACCAG	61 bp
**Adapter sequence Forward**	TCGTCGGCAGCGTCAGATGTGTATAAGAGACAG	67 bp
**Adapter sequence Forward**	GTCTCGTGGGCTCGGAGATGTGTATAAGAGACAG	67 bp

Supplementary Tables:

Table S1: Information regarding mutations that were reported so far in *KIZ*.

c.	p.	Exon	Publication/s	Number of affected (homozygous, compound heterozygous)
c.3G>A	p.M1?	1	The current study<br>[1]	2 (0,2)<br>1 (1,0)
c.52G>T	p.E18*	1	[2]	1 (0,1)
c.86_89+19del	p.R28_S30del	1	Submitted in ClinVar<br>Accession: VCV000813294.1<br>Variation ID:813294	1(1,0)
c.119_122delAACT	p.K40Ifs*14	2	[2]<br>[3]<br>[4]	1 (0,1)<br>1 (1,0)<br>1 (1,0)
c.226C>T	p.R76*	3	The current study<br>[2]<br>[5]<br>[1]<br>[3]<br>[6]<br>[4]<br>[7]<br>[8]	23 (21,2)<br>2 (2,0)<br>2 (2,0)<br>1 (1,0)<br>2 (2,0)<br>7 (7,0)<br>3 (3,0)<br>1 (1,0)<br>1 (1,0)
c.247C>T	p.R83*	3	The current study and<br>Submitted in ClinVar.<br>Accession: VCV001933998.2	1 (1, 0)
c.251_258del	p.F84Cfs*23		[9]	1 (1,0)
c.583C>T	p.R195*	5	ClinVar: RCV001075798.1<br>[10]- Heterozygous	1 (1,0)

Table S2: Clinical information of 25 patients with biallelic *KIZ* pathogenic variants.

Patient number (age of onset)	Age of clinical testing	Visual Acuity	Refraction (in D)	Cone flicker 30Hz (μV;msec)[d]	Mixed cone-rod (a wave, b wave in μV)	Rod response (μV)	EOG
MOL0289-3 (18)	25<br>26<br>27<br>29<br>31<br>33	0.62<br>0.50<br>0.52<br>0.45<br>0.42<br>0.41					
MOL0336-1 (35)	36<br>37	0.37<br>0.58	-0.75	ND	61, 115	35	
MOL0445-1 (childhood)	23<br>29	1.00<br>0.74	plano				
MOL0610-1 (32)	35<br>38	0.99<br>0.91		 85 (32.9)	 127, 200	 139	 147
MOL0845-1 (23)	27<br>31<br>34<br>44<br>56<br>56<br>58<br>61<br>63<br>65<br>66	0.40<br><br>0.58<br>0.25<br>0.40<br>HM<br>HM<br>HM<br>0.001<br>HM<br>0.0005		 SR	 SR	 SR	 120
MOL1015-1	62	0.08	-2.00				

(40)							
MOL1156-1<br>(20)	49	0.02	-5.00				
MOL1440-1	44	0.34					
MOL1440-2<br>(childhood)	43	0.33	+3.00				
MOL1523-1	62<br>63<br>66	0.62<br>0.75<br>0.75	 <br>+6.50	54 (30)	140, 254	178	177
MOL1605-1	13<br>14<br>15<br>16<br>17<br>19	0.50<br>0.53<br>0.45<br>0.34<br>0.26<br>0.35	 <br><br><br><br>-0.5	 26 (38.5)	 39, 57	 ND	 117
MOL1621-1<br>(3rd decade)	42	0.33					
MOL1663-1<br>(45)	45<br>56<br>58<br>60<br>61	 0.63<br>0.89<br>0.50<br>0.65		76 (38.9)	98, 147	65	110
MOL1684-1<br>(20)	30<br>56<br>58	 <br>HM/FC<br>30cm		16 (43.5)	ND	ND	
MOL1689-1<br>(20)	21<br>23	0.69<br>0.79		54 (34)	74, 150	124	159
MOL1720-1<br>(4th decade)	50	0.50		39 (33)	60, 98	64	
MOL1819-1	50	0.99	-2.20				

(4th decade)							
TB240- R565<br>(18)	18<br>22<br>36	 0.75<br>0.80	 -.0.50	ND	ND	ND	
TB338- R721	57	0.16					
TB675-<br>R1228<br>(18)	22	0.9					
TB711-<br>R1291	46	0.90					
TB736-<br>R1328	43	0.75	-0.50	46 (34.5)	70, 99	39	
TB928-<br>R1586	39<br><br>47	0.1/<br>HM 1m<br>0.05		ND	ND	ND	
TB980-<br>R1667	31<br>61	0.10<br>HM	-0.50	SR	40	65	
TB1212-<br>R1970<br>(45)	65<br>75	0.91<br>0.60	+0.75	62 (33)	123, 208	137	
**A list of previously reported cases with published clinical data**							
**Patient number (age of onset)**	**Age of clinical testing**	**Visual Acuity**	**Refraction**	**Cone flicker 30Hz (µV;msec)[d]**	**Mixed cone-rod (a wave, b wave in µV)**	**Rod response (µV)**	**Reference**
CIC00173	50	0.03		ND	ND	ND	[2]
CIC01611	34	1		ND	ND	ND	[2]
CIC01225	51	0.57		ND	ND	ND	[2]

II:2/F	26	0.025					[5]
II:4/F	44	0.8					[5]
A-II:2	62	0.57		19%		ND	[4]
B-II:1	60	0.09				42	[4]
C-II:1	21	0.73					[4]
D-II:5	35	0.01		19%		ND	[4]

SR- severely reduced, ND- non-detectable, HM-hand motion, FC- finger counting, D- dioptre

Table S3: The seven principal components sample variances obtained in this study.

	PC1	PC2	PC3	PC4	PC5	PC6	PC7
sample variance	0.673	0.129	0.089	0.055	0.039	0.012	0.003

Table S4: The variable coefficient in each of the seven principal component.

	PC1	PC2	PC3	PC4	PC5	PC6	PC7
Disease onset age	0.32	0.65	0.09	-0.14	-0.67	-0.02	0.01
Age of presentation	0.34	0.59	0.01	0.17	0.70	0.08	0.00
Rod response	0.41	-0.22	-0.11	-0.60	0.08	0.56	0.30
Cone flicker amplitude	0.38	-0.20	-0.35	0.70	-0.22	0.39	-0.03
a-wave cone-rod responses	0.44	-0.19	-0.16	0.03	0.02	-0.68	-0.53
b-wave cone-rod responses	0.44	-0.20	-0.13	-0.23	0.05	-0.26	-0.79
BCVA slop	0.30	-0.24	0.90	0.18	-0.01	0.06	0.01

Patient score can be calculated for each principal component and patient by multiplying each variable coefficient in this table with its variable value within the particular patient and summing all 7 products within this patient.

Table S5: information regarding *KIZ* transcript.

Transcript Name	Transcript Description	Gel Densitometry (Mean ± SD)		Percent of Reads by NGS Analysis (Mean ± SD)	
		**Controls**	**Patients**	**Controls**	**Patients**
**3A**: List of KIZ transcripts that were included in the current analysis					
NM_018474.6	Canonical	19.4 ± 4.2%	7.5 ± 1.3%	51.6 ± 5.1 %	21.1 ± 5.1%
NM_001163022.3	Skipping of exon 3	11.5 ± 1.2%	18.0 ± 8.0%	12.2 ± 3.0 %	26.3 ± 4.4%
NM_001163023.3	Skipping of exons 3 and 4	4.2 ± 1.0%	6.8 ± 2.2%	36.5 ± 9.6%	52.3 ± 1.2%
NM_001352436.2	skipping exon 3 and carrying an additional exon between exon 3 and 4	**Not Applicable**		0.2 ± 0.1%	0.3 ± 0.1%
**3B:** A list of additional KIZ transcripts that were not included in the current study					
NM_001276389.2	Skipping of exons 2, 3 and 4	**Not Applicable**		**Not Applicable**	
NM_001352434.2	missing a part of exon 7				

The table summarizes the *KIZ* transcript information from a total of four controls and three patient's fibroblasts that were included in the study. The values presented in this table for each transcript represents the mean standard deviation from four controls and three patients.

Figure S1: Pedigrees of *KIZ*-related families

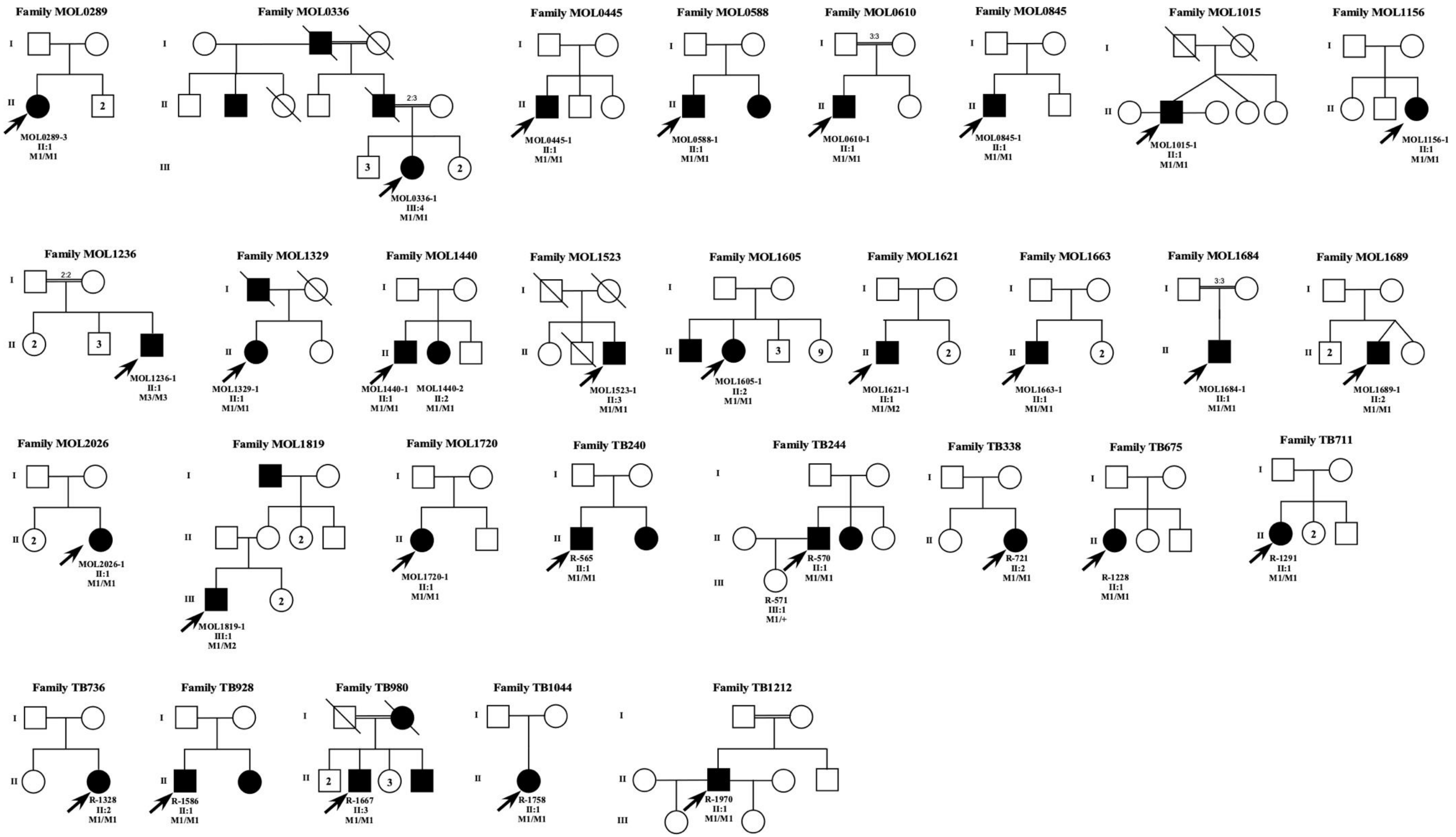

Figure S2: Comparison of clinical parameters by two-sided t-test.

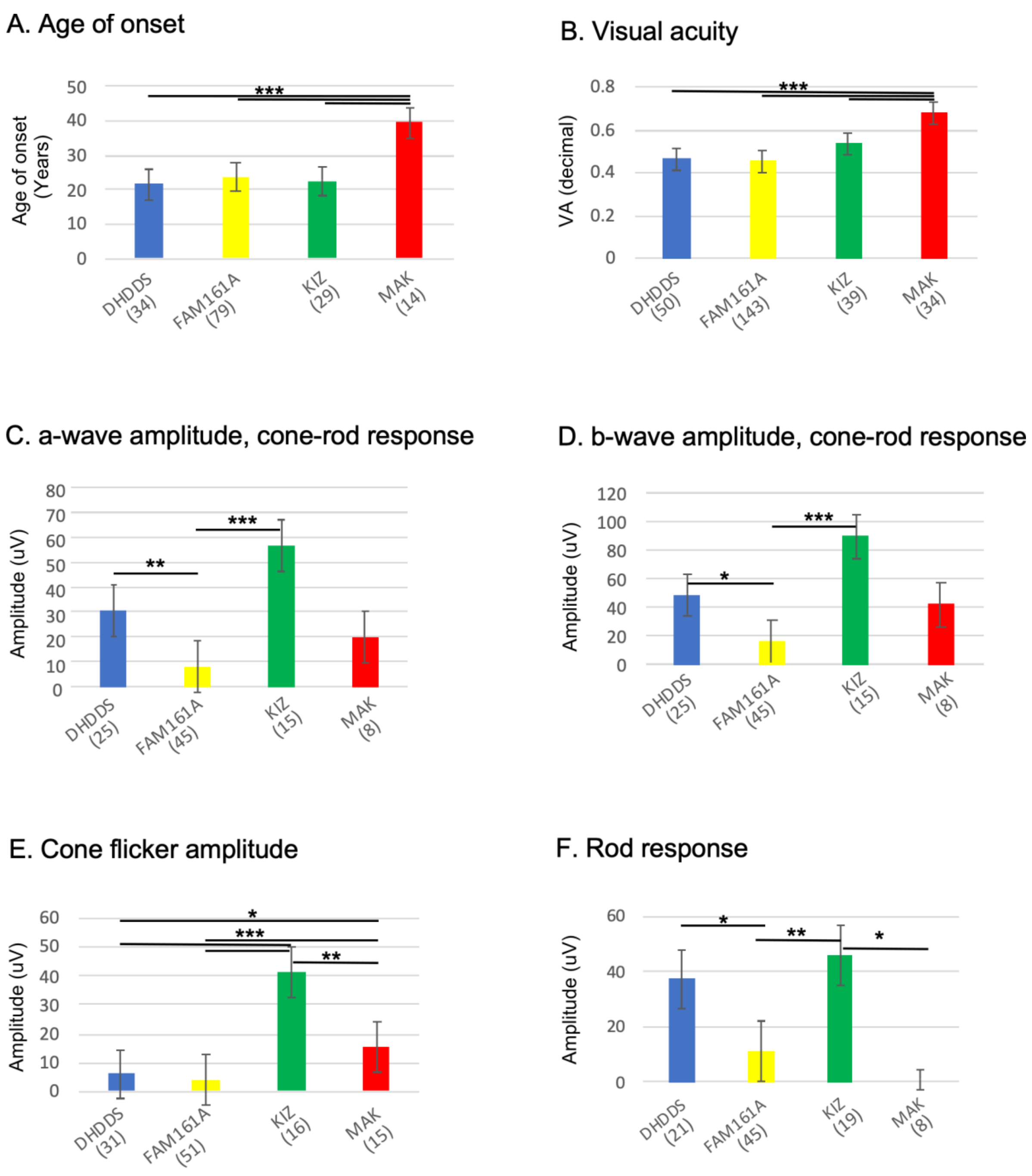

For each clinical parameter, the average ± SD are presented for each gene. The number of point data for each gene are in parenthesis. Statistical analysis was done using the t-test analysis. P values are indicated as * (0.01-0.05), ** (0.01-0.001), and *** (<0.001).

Figure S3: EOG versus ERG data

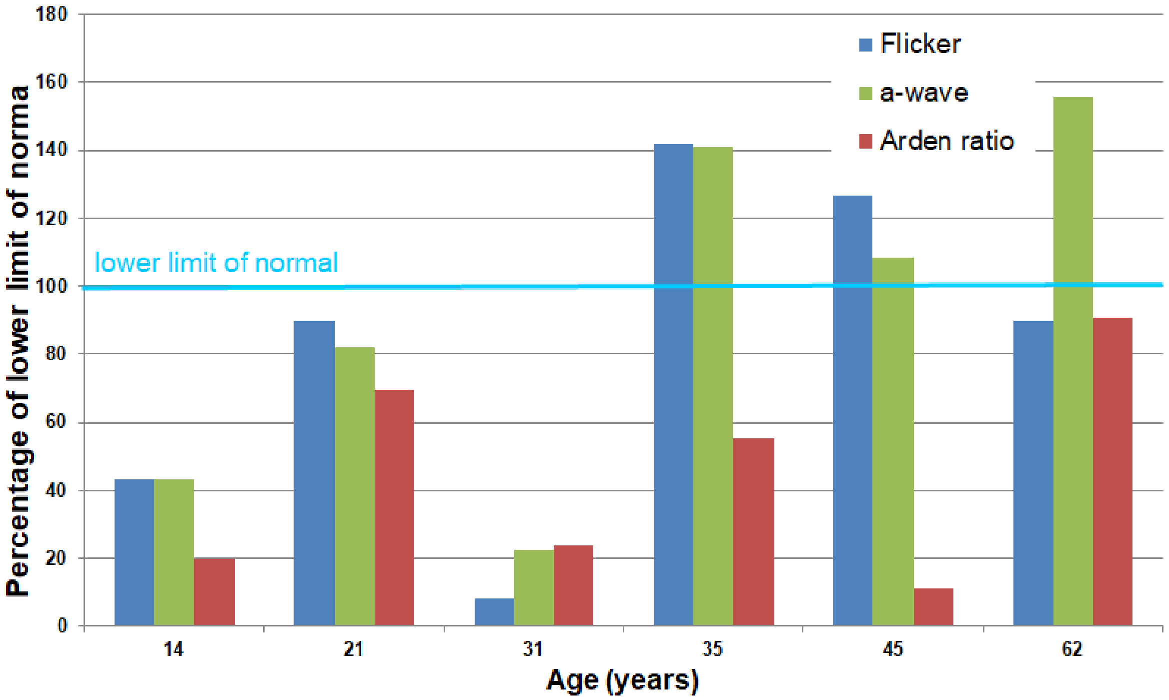

The bar graph presents the percentage compared to the lower limit of normal values of cone flicker amplitude (blue bars), a-wave of the mixed response (green bars) and Arden ratio (EOG- red bars) of six patients with biallelic *KIZ* mutations between ages 14 and 62 years. The 100% line represents the lower limit of normal response and therefore values can either be above this value and are considered “within normal range” of below this value. All patients showed recordable responses, even at older ages and most of them with normal or near normal amplitudes.

Figure S4: RT-PCR analysis of normal mice and sheep retina

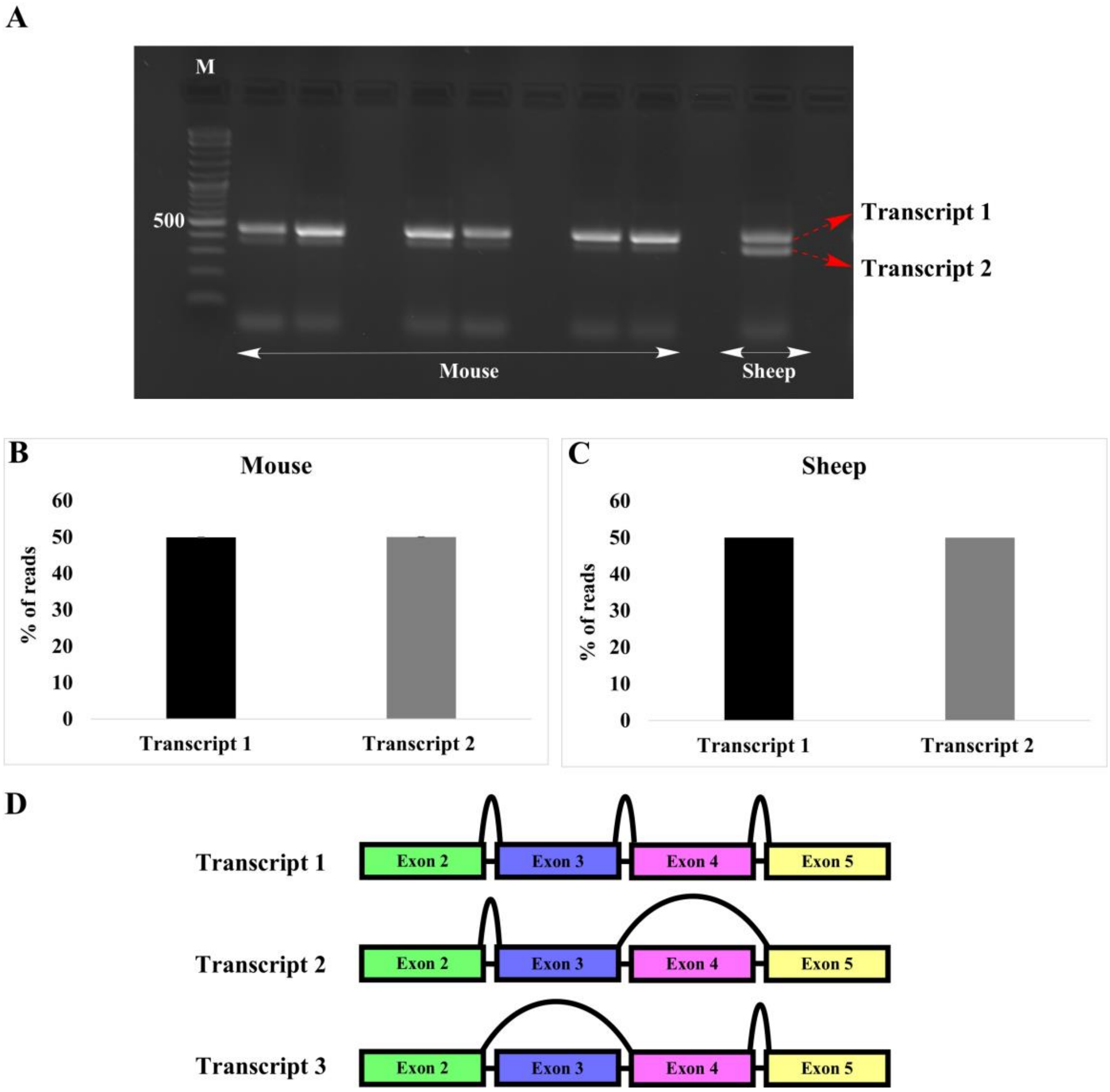

RT-PCR analysis on RNA isolated from normal mice and sheep retina identified two different transcript (A). NGS analysis revealed no difference in the percentage of reads between the transcripts in both the species (B and C). A small fraction of transcripts (0.10%) skipped exon 3 in mice retina (D).

Figure S5: Ciliary analysis of c.226C>T fibroblasts

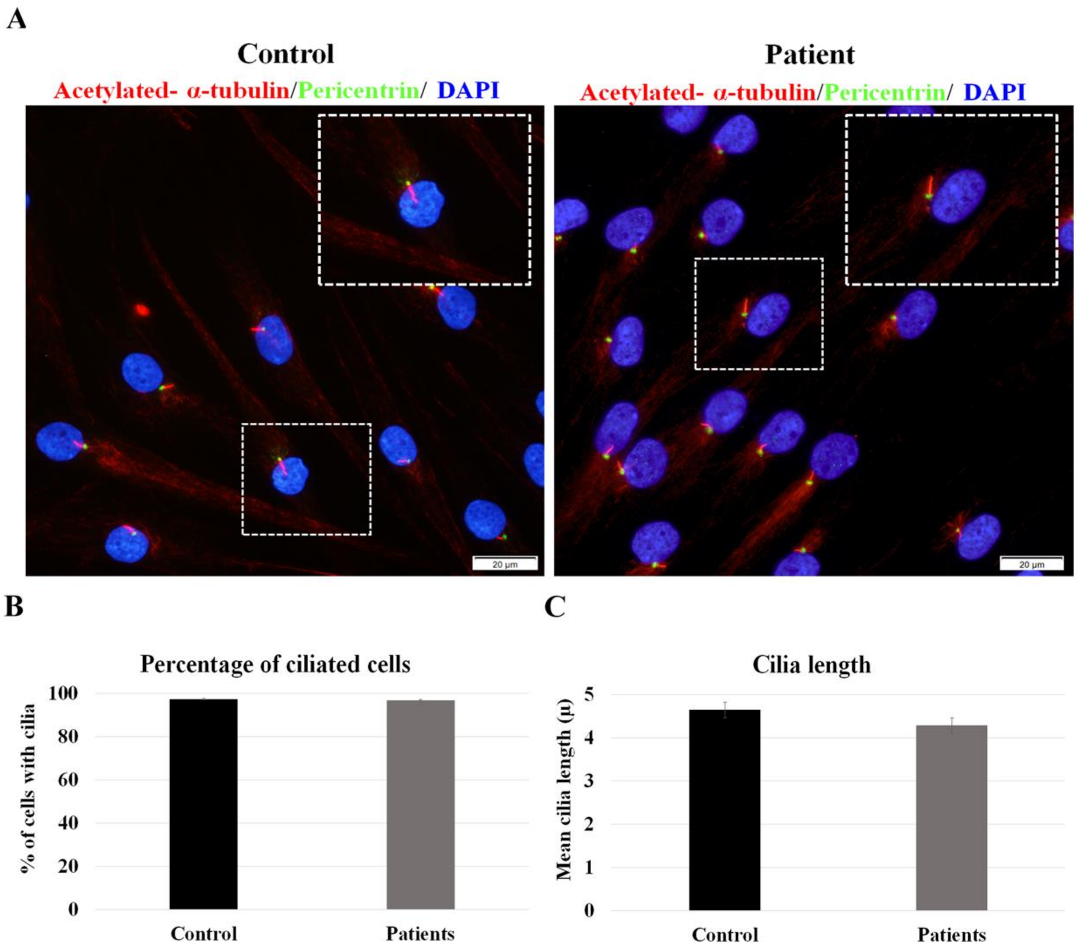

Fibroblasts (n=100) from controls and patients were immunostained for acetylated-α-tubulin (red) and pericentrin (green) to determine the effect of the mutation c.226C>T (A). No difference was evident between the percentage of ciliated cells (B) as well as the length of cilia (C) between the groups.

References

1. El Shamieh, S.; Méjécase, C.; Bertelli, M.; Terray, A.; Michiels, C.; Condroyer, C.; Fouquet, S.; Sadoun, M.; Clérin, E.; Liu, B.; et al. Further Insights into the Ciliary Gene and Protein KIZ and Its Murine Ortholog PLK1S1 Mutated in Rod-Cone Dystrophy. *Genes (Basel)* **2017**, *8*, doi:10.3390/genes8100277.
2. El Shamieh, S.; Neuillé, M.; Terray, A.; Orhan, E.; Condroyer, C.; Démontant, V.; Michiels, C.; Antonio, A.; Boyard, F.; Lancelot, M.-E.; et al. Whole-Exome Sequencing Identifies KIZ as a Ciliary Gene Associated with Autosomal-Recessive Rod-Cone Dystrophy. *Am J Hum Genet* **2014**, *94*, 625–633, doi:10.1016/j.ajhg.2014.03.005.
3. Jauregui, R.; Chan, L.; Oh, J.K.; Cho, A.; Sparrow, J.R.; Tsang, S.H. Disease Asymmetry and Hyperautofluorescent Ring Shape in Retinitis Pigmentosa Patients. *Sci Rep* **2020**, *10*, 3364, doi:10.1038/s41598-020-60137-9.
4. Lin, Y.; Xu, C.L.; Breazzano, M.P.; Tanaka, A.J.; Ryu, J.; Levi, S.R.; Yao, K.; Sparrow, J.R.; Tsang, S.H. Progressive RPE Atrophy and Photoreceptor Death in KIZ-Associated Autosomal Recessive Retinitis Pigmentosa. *Ophthalmic Genet* **2020**, *41*, 26–30, doi:10.1080/13816810.2020.1723116.
5. Gustafson, K.; Duncan, J.L.; Biswas, P.; Soto-Hermida, A.; Matsui, H.; Jakubosky, D.; Suk, J.; Telenti, A.; Frazer, K.A.; Ayyagari, R. Whole Genome Sequencing Revealed Mutations in Two Independent Genes as the Underlying Cause of Retinal Degeneration in an Ashkenazi Jewish Pedigree. *Genes (Basel)* **2017**, *8*, doi:10.3390/genes8090210.
6. Sharon, D.; Ben-Yosef, T.; Goldenberg- Cohen, N.; Pras, E.; Gradstein, L.; Soudry, S.; Mezer, E.; Zur, D.; Abbasi, A.H.; Zeitz, C.; et al. A Nation-wide Genetic Analysis of Inherited Retinal Diseases in Israel as Assessed by the Israeli Inherited Retinal Disease Consortium (IIRDC). *Hum Mutat* **2019**, *41*, 140–149, doi:10.1002/humu.23903.
7. Villafuerte-de la Cruz, R.A.; Garza-Garza, L.A.; Garza-Leon, M.; Rodriguez-De la Torre, C.; Parra-Bernal, C.; Vazquez-Camas, I.; Ramos-Gonzalez, D.; Rangel-Padilla, A.; Espino Barros-Palau, A.; Nava-García, J.; et al. Spectrum of Variants Associated with Inherited Retinal Dystrophies in Northeast Mexico. *BMC Ophthalmol* **2024**, *24*, 60, doi:10.1186/s12886-023-03276-7.
8. Zhao, Y.; Coussa, R.G.; DeBenedictis, M.J.M.; Traboulsi, E.I. Retinal Dystrophy Associated with a Kizuna (KIZ) Mutation and a Predominantly Macular Phenotype. *Ophthalmic Genet* **2019**, *40*, 455–460, doi:10.1080/13816810.2019.1666880.
9. Weisschuh, N.; Obermaier, C.D.; Battke, F.; Bernd, A.; Kuehlewein, L.; Nasser, F.; Zobor, D.; Zrenner, E.; Weber, E.; Wissinger, B.; et al. Genetic Architecture of Inherited Retinal Degeneration in Germany: A Large Cohort Study from a Single Diagnostic Center over a 9-Year Period. *Hum Mutat* **2020**, *41*, 1514–1527, doi:10.1002/humu.24064.

10. Méjécase, C.; Kozak, I.; Moosajee, M. The Genetic Landscape of Inherited Eye Disorders in 74 Consecutive Families from the United Arab Emirates. *Am J Med Genet C Semin Med Genet* **2020**, *184*, 762–772, doi:10.1002/ajmg.c.31824.
